# ﻿Notes on *Strobilanthes* (Acanthaceae) with capitate inflorescences in Thailand

**DOI:** 10.3897/phytokeys.244.124260

**Published:** 2024-07-09

**Authors:** Pornchai Kladwong, Pranom Chantaranothai

**Affiliations:** 1 Department of Forest Biology, Faculty of Forestry, Kasetsart University, Bangkok 10900, Thailand Kasetsart University Bangkok Thailand; 2 Department of Biology, Center of Excellence on Biodiversity (BDC) and Applied Taxonomic Research Center (ATRC), Faculty of Science, Khon Kaen University, Khon Kaen 40002, Thailand Khon Kaen University Khon Kaen Thailand; 3 Royal Botanic Gardens, Kew, Richmond, Surrey, TW9 3AE, UK Royal Botanic Gardens Richmond United Kingdom

**Keywords:** Identification key, new record, new species, nomenclature

## Abstract

Twenty-three species of *Strobilanthes* Blume with capitate inflorescences are enumerated in Thailand. *Strobilanthesphengklaii* Kladwong & Chantar., a new species from North-eastern Thailand, is described and illustrated. *Strobilanthespaniculiformis* J.R.I.Wood, *S.phyllostachya* Kurz and *S.squalens* S.Moore are new records in Thailand. *Strobilantheskerrii* Craib is reinstated, and S.evrardiivar.parviflora J.B.Imlay, *S.bombycina* J.B.Imlay, *Hemigraphishispidula* Craib and *Sericocalyxthailandicus* Bremek. are treated as new synonyms. 19 taxa are lectotypified. A key to species, taxonomic notes and photographs are provided as well as a preliminary conservation assessment and distribution maps.

## ﻿Introduction

*Strobilanthes* Blume is a genus of Acanthaceae consisting of ca. 454 species mostly distributed in tropical and subtropical Asia (Mabberley 2008; Christenhusz et al. 2017; Tripp et al. 2021; POWO 2023; WFO 2024). *Strobilanthes* species are herbs, subshrubs, shrubs or small trees, rarely plants are creeping. Many species are gregarious, and some have a plietesial life cycle, living several years before flowering and then, once having flowered, dying (Beentje 2016). This genus is unique in having the rugula and trichomes retaining the style (Fig. 6F) on the inner surface of posterior corolla (Wood, 1994b; Carine and Scotland 2002; Moylan et al. 2004; Wood and Scotland 2009; Hu et al. 2011; Tripp et al. 2021; Kladwong and Chantaranothai 2022).

Furthermore, three inflorescence types, spicate, paniculate and capitate are found in *Strobilanthes* species. This informal grouping as used by Wood and Scotland (2003b), Hu et al. (2011) and Kladwong and Chantaranothai (2023) but is not used in other treatments, e.g., Bremekamp (1944), Wood (1994b). The informal grouping may not work throughout the range of *Strobilanthes*, but it does provide a robust way of identifying Thai species. In the capitate type the flowers are clustered towards the tips of the strongly abbreviated inflorescence axes, and the involucral bracts and flowers are densely arranged. The bracts are variable in size and shape with the outer bracts generally larger than the inner ones (Bremekamp 1944; Bennett and Scotland 2003; Wood and Scotland 2003b; Hu et al. 2011; Kladwong and Chantaranothai 2023). A species list of all three groups based on inflorescence types reported in Thailand is presented in Table 1.

**Table 1.** List of species in the three groups of inflorescence types present in Thailand.

Paniculate inflorescences ^a, b^	Capitate inflorescences	Spicate inflorescences
1. *S.microcarpa* T.Anderson	1. *S.aprica*	1. *S.abbreviata* Y.F.Deng & J.R.I.Wood
2. *S.pedunculosa* Miq.	2. *S.articulata*	2. *S.alboviridis* J.B.Imlay
3. *S.peninsularis* Terao	3. *S.brandisii*	3. *S.alternata* (Burm.f.) Moylan ex J.R.I.Wood
4. *S.tenuiflora* J.R.I.Wood	4. *S.capitata*	4. *S.argentea* J.B.Imlay
5. *S.trichantha* J.R.I.Wood	5. *S.chiangdaoensis*	5. *S.auriculata* Nees
6. *S.violifolia* T.Anderson	6. *S.consors*	6. *S.bilabiata* J.R.I.Wood
	7. *S.cruciata*	7. *S.collina* Nees
	8. S.dimorphotrichasubsp.rex	8. *S.corrugata* J.B.Imlay
	9. *S.echinata*	9. *S.cusia* (Nees) Kuntze
	10. *S.erecta*	10. *S.dalzielii* (W.W.Sm.) Benoist
	11. *S.esquirolii*	11. *S.decumbens* (Bremek.) J.R.I.Wood
	12. *S.falconeri*	12. *S.fluviatilis* (C.B.Clarke ex W.W.Sm.) Moylan & Y.F.Deng
	13. *S.graminea*	13. *S.fragrans* J.R.I.Wood
	14. *S.hypomalla*	14. *S.glaucescens* Wall. ex Nees
	15. *S.kerrii*	15. *S.heliophila* J.R.I.Wood
	16. *S.paniculata*	16. *S.hossei* C.B.Clarke
	17. *S.paniculiformis*	17. *S.imbricata* Nees
	18. *S.phengklaii*	18. *S.karensium* Kurz
	19. *S.phyllocephala*	19. *S.maxwellii* J.R.I.Wood
	20. *S.phyllostachya*	20. *S.moschifera* Blume
	21. *S.serpens*	21. *S.pateriformis* Lindau
	22. *S.speciosa*	22. *S.quadrifaria* (Wall. ex Nees) Y.F.Deng
	23. *S.squalens*	23. *S.ranongensis* Terao
		24. *S.repanda* (Blume) J.R.Benn.
		25. *S.reptans* (G.Forst.) Moylan ex Y.F.Deng & J.R.I.Wood
		26. *S.rivularis* J.R.I.Wood & J.R.Benn.
		27. S.rufescenssubsp.parishii (C.B.Clarke) J.R.I.Wood subsp. parvibracteata (C.B.Clarke) J.R.I.Wood
		28. *S.schomburgkii* (Craib) J.R.I.Wood
		29. *S.serrata* J.B.Imlay
		30. *S.tonkinensis* Lindau
		31. *S.violascens* Ridl.
		32. *S.xanthosticta* C.B.Clarke

^a^ Wood and Scotland (2003); ^b^Kladwong and Chantaranothai (2023).

During the preparation of the taxonomic account of *Strobilanthes* for the Flora of Thailand we recently reported six Thai *Strobilanthes* species with paniculate inflorescences (Kladwong and Chantaranothai 2023). Taxonomic work on the spicate species is ongoing and this paper enumerates the 23 native species and one subspecies of the capitate group in Thailand. Six species are endemic in Thailand. A new species of *Strobilanthes* is described and three species are newly recorded. The identification key and nomenclatural treatments are presented.

## ﻿Material and methods

This taxonomic study is based on extensively field observations in Thailand and the investigation of herbarium specimens at AAU, BK, BKF, BM, CMU, CMUB, E, E-GL, K, K-W, KKU, KYO, L, PSU, QBG, TCD and U. Additionally, specimens from A, ABD, B, C, CAL, CORD, G, GH, GZU, M, NY, P and SING were studied from digital images since they are available on each herbarium website and the GBIF (https://gbif.org) website. Herbarium citations follow Thiers (2021). The nomenclature follows Turland et al. (2018), and binomial authorities follow IPNI (2023). Relevant literature including Hosseus (1907), Craib (1911, 1912, 1913, 1914), Benoist (1935), Imlay (1938, 1939), Bremekamp (1944, 1953, 1961, 1965, 1966, 1969), Terao (1980, 1981, 1983), Hansen (1985), Wood (1994b), Bennett and Scotland (2003), Wood and Scotland (2003b, 2009), Hu et al. (2011), Albertson and Wood (2012), Pooma and Suddee (2014), Newman et al. (2007, 2017), Wood et al. (2022) and Kladwong and Chantaranothai (2022, 2023) were consulted. Global conservation assessments were made using the categories and criteria of IUCN (2022); EOO and AOO were calculated using GeoCAT (Bachman et al. 2011).

## ﻿Taxonomic treatment

### ﻿Notes on taxonomic characters

The unequal leaf pairs are observed in many species of *Strobilanthes* (Wood 1994b; Hu et al. 2011). This character is variable in some species, but it can be used to group *S.articulata*, *S.phyllocephala* and *S.falconeri*. The bracts are commonly used to distinguish species, especially using shapes, sizes and indumentum types (Clarke 1884; Bremekamp 1944; Wood 1994b; Bennett and Scotland 2003; Hu et al. 2011). Moreover, the occurrence of leaf-like bracts or the bract having a petiolar base (Bremekamp 1944; Wood and Scotland 2009; Hu et al. 2011) are also used to recognize the species such as *S.brandisii*, *S.consors*, *S.cruciata*, *S.erecta*, *S.esquirolii*, *S.falconeri* and *S.phyllocephala*. The calyx is useful for taxonomic delimitation (Nees 1832; Bremekamp 1944; Wood and Scotland 2003a; Hu et al. 2011). The calyx lobes are subequal in almost all species whereas they have one lobe longer than others in *S.dimorphotricha* and *S.paniculiformis*. The colour of corolla is usually purple or pale purple or mauve or pale blue, but rarely white or yellow (Benoist 1935; Imlay 1938, 1939; Bennett and Scotland 2003). The white corolla is found only in *S.cruciata*. Purple and white can occur in species such as *S.kerrii* and *S.speciosa*. The yellow corolla is rare, and this character is observed in *S.phengklaii*, *S.squalens* and *S.phyllostachya*.

### ﻿Conservation assessments

23 species of *Strobilanthes* with capitate inflorescences are enumerated in Thailand. Three species, *S.chiangdaoensis*, *S.paniculiformis* and *S.phyllostachya* are assessed as vulnerable. Two species, *S.graminea* and *S.hypomalla* are endangered. All localities of the threatened species are in protected areas such as the national park and wildlife sanctuary, but they have only a few records. Moreover, they also need specific habitats such as the rugged limestone ridge in mixed evergreen and deciduous forests or the open plateau of evergreen mountains and the sandy soil in dipterocarp forest. The changes of habitats are occurring through increasing droughts and fires caused by humans leading to the decline of the threatened species. *Strobilantheschiangdaoensis* and *S.phyllostachya* are cultivated at Queen Sirikit Botanic Garden. *Strobilanthesarticulata* and *S.phengklaii*, *S.phyllocephala* are Data Deficient with few collections; more field work is needed to assess these species. Further details on conservation assessments of the threatened species are provided under the species accounts below.

### ﻿Key to the species of *Strobilanthes* with capitate inflorescences in Thailand

**Table:** 

1	Flowers pedicellate, separately arranged into panicles	**Paniculate inflorescence group**
–	Flowers sessile or subsessile, lax or densely arranged	**2**
2	Flowers arranged along inflorescence axes; bracts and flowers lax or densely arranged into spikes; bracts arranged into lower and upper ones, lower and upper bracts same size or the lower bracts generally larger than the upper ones	**Spicate inflorescence group**
–	Flowers clustered towards the tips of the inflorescence axes; bracts and flowers densely arranged into terminal heads; bracts arranged into outer and inner ones, outer bracts generally larger than the inner ones	**Capitate inflorescence group 3**
3	Leaf margin entire or subentire except serrulate in *S.aprica*; stamens 2, exserted	**4**
–	Leaf margin serrate, crenate or dentate; stamens 4, included except *S.paniculata*	**6**
4	Capsule 8-seeded	**13. *S.graminea***
–	Capsule 4-seeded	**5**
5	Leaves lanceolate to oblong-lanceolate; leaf margin entire; corolla pubescent outside	**14. *S.hypomalla***
–	Leaves elliptic to oblong-elliptic; leaf margin serrulate; corolla glabrous or subglabrous outside	**1. *S.aprica***
6	Corolla yellow	**7**
–	Corolla white or purple or whitish purple or pale purple or mauve or pale blue or whitish-cream or pale violet	**9**
7	Leaves lanceolate to oblong-lanceolate; bracts densely sericeous	**18. *S.phengklaii***
–	Leaves obovate to obovate-oblong; bracts puberulous or hirsute	**8**
8	Bracts lanceolate to linear-lanceolate, acute at apex; corolla sparsely hairy outside	**23. *S.squalens***
–	Bracts elliptic, ligulate at apex; corolla glabrous outside	**20. *S.phyllostachya***
9	Stamens exserted	**17. *S.paniculata***
–	Stamens included	**10**
10	Capsules 8-seeded	**21. *S.serpens***
–	Capsules 2-seeded or 4-seeded	**11**
11	Capsules 2-seeded	**5. *S.chiangdaoensis***
–	Capsules 4-seeded	**12**
12	Outer bracts leaf-like or with petiolar base	**13**
–	Outer bracts ovate to orbicular or ovate or obovate to spatulate or elliptic-lanceolate or lanceolate to linear-lanceolate or oblanceolate, sessile	**19**
13	Corolla white; ovary glabrous	**7. *S.cruciata***
–	Corolla whitish-purple or blue; ovary hairy at apex	**14**
14	Bracts, bracteoles and calyx densely white tomentose	**15**
–	Bracts, bracteoles and calyx hirsute or pilose or glabrous	**17**
15	Stems sulcate, dark green; bracts oblong-lanceolate, dark green	**11. *S.esquirolii***
–	Stems not sulcate, yellowish-green; bracts obovate or oblanceolate or spathulate, yellowish-green or whitish-green	**16**
16	Heads ellipsoid; bracts curved; bracteoles acute to acuminate at apex; calyx lobes acuminate at apex	**6. *S.consors***
–	Heads suborbicular; bracts flat; bracteoles obtuse at apex; calyx lobes acute at apex	**3. *S.brandisii***
17	Plants isophyllous or subisophyllous	**10. *S.erecta***
–	Plants anisophyllous	**18**
18	Stems villose or tomentose; leaves elliptic-lanceolate or lanceolate; bracteole acute at apex	**12. *S.falconeri***
–	Stems pubescent or glabrescent; leaves ovate or ovate-elliptic; bracteoles obtuse at apex	**19. *S.phyllocephala***
19	Inflorescence axis very slender; bracteoles absent	**2. *S.articulata***
–	Inflorescence axis not as above; bracteoles present	**20**
20	Bracteoles and calyx dentate or fimbriate or dentate-crenate at apex	**9. *S.echinata***
–	Bracteoles and calyx rounded or obtuse or acute to acuminate	**21**
21	Calyx lobes with 1 lobe longer than others	**22**
–	Calyx lobes subequal	**23**
22	Bracts ovate or elliptic-lanceolate; bracteoles lanceolate to ovate-lanceolate, acute at apex	**8. S.dimorphotrichasubsp.rex**
–	Bracts ovate-orbicular; bracteoles obovate to oblong-oblanceolate, rounded at apex	**16. *S.paniculiformis***
23	Leaf pairs weakly unequal, similar in shape; bracts curved; corolla bluish to purplish blue	**4. *S.capitata***
–	Leaf pairs strongly unequal, differ in shape; bracts flat; corolla purplish or white	**24**
24	Stems, petiole and peduncle with purplish hairs; smaller lamina elliptic or suborbicular-ovate	**15. *S.kerrii***
–	Stems, petiole and peduncle without purplish hairs; smaller lamina lanceolate to linear-lanceolate	**22. *S.speciosa***

#### ﻿1. Strobilanthes aprica

Taxon classificationPlantaeLamialesAcanthaceae

(Hance) T.Anderson ex Benth., Fl. Hongk. 262. 1861.

CDB2D128-ABAB-516E-A914-868128437B15

Fig. 10A

Gutzlaffia aprica Hance, Hooker’s J. Bot. Kew Gard. Misc. 1: 142. 1849. Type: China, Hong Kong, *Hance 536* (lectotype CAL [CAL0000019794 image!] designated by Albertson and Wood 2012, pg. 50; isolectotype GH [GH00387581 image!]).
Strobilanthes aprica var. glabra J.B.Imlay, Bull. Misc. Inform. Kew 1939(3): 116. 1939. Type: Thailand, Lamphun [Lampun]; Mae Kaw, 9 Sept 1924, *Winit 1231* (holotype ABD [ABDUH:2/885 image!]; isotypes BK [257642!], BKF [SN001358!]).
Gutzlaffia pedunculata Craib, Bull. Misc. Inform. Kew 1911(10): 436. 1911. Type: Thailand, Chiang Mai, Doi Suthep [Doi Sootep], 25 Sept 1910, *Kerr 1430* (lectotype K [K001514863!] designated here; isolectotypes BM [BM000796839!], C [C10005192 image!], K [K001514864!], L [L2832219!], P [P00719397 image!]).
Strobilanthes aprica var. pedunculosa (Craib) Benoist in Lecomte et al., Fl. Indo-Chine 4: 666. 1935. Type: Based on Gutzlaffiapedunculata Craib

##### Type.

Based on *Gutzlaffiaaprica* Hance

##### Distribution.

Myanmar, China, Taiwan, Thailand, Laos, Vietnam, Cambodia.

##### Ecology.

On open limestone hill or in open pine forest, open dipterocarp forest, open evergreen forest and open sandy grassland; 240–1,975 m alt, flowering and fruiting from August to April.

##### Selected specimens examined.

Thailand, Northern: **Mae Hong Son**, Khun Yuam, 650 m alt., 14 Jan 1988, *Santisuk 6671* (BKF); ibid., Mae La Noi, 430 m alt., 27 Dec 1965, *Hennipman 3494* (BKF); **Chiang Mai**, Doi Chiang Dao WS, 21 Dec 1931, *Put 4460* (BK, BM, K); ibid., Doi Pui, Huai Hee, 1,600 m alt., 22 Oct 2000, *Suksathan 2815* (QBG); ibid., 15 Oct 2019, Kiw Lom, *Kladwong 495* (KKU) & *496* (KKU); ibid., Doi Suthep NP, 25 Sept 1910, *Kerr 1430* (BM, K, L, P); ibid., 1,500 m alt., 11 Nov 1973, *Smitinand 11844* (BKF); ibid., 850 m alt., 1 Oct 1985, *Sørensen* et al. *5378* (BKF, E); ibid., 450 m alt., 12 Dec 1907, *Maxwell 87-1586* (AAU, BKF); **Lamphun**, Mae Kaw, 430 m alt., 9 Sept 1924, *Winit 1231* (ABD, BK, BKF); **Lampang**, Pa Tat, Pe Tra, 360 m alt., 13 Dec 1926, *Winit 1815* (AAU, BK, BKF); **Tak**, Tha Song Yang, Khao Hua Mot Noi, 5 km before Ban Tha Song Yang, 160 m alt., 23 Dec 2010, *Suksathan* et al. *5375* (L). North-eastern: **Phetchabun**, Nam Nao NP, Pha Daeng Cliff, 900–959 m alt., 26 Dec 1982, *Koyama* et al. *31730* (BKF, KYO); **Loei**, Phu Kradueng NP, 15 Aug 1946, *Din 189* (BKF); ibid., 1,300 m alt., 10 Nov 1976, *Smitinand 12221* (BKF). South-western: **Kanchanaburi**, Khao Meng, 14 Apr 1965, *Chantanamuck 1061* (BK).

##### Preliminary conservation status assessment.

This species has an Extent of Occurrence (EOO) of 121,951.239 km^2^ and an Area of Occupancy (AOO) of 56.000 km^2^ and is assessed as Least Concern (LC) following IUCN (2022).

##### Notes.

*Strobilanthesaprica* is similar to *S.graminea* J.B.Imlay in having a gibbose and curved corolla, glabrous or subglabrous outside and 2 exserted stamens. It can be distinguished based on elliptic or oblong-elliptic to lanceolate leaf and hairy bract and bracteole vs. oblong-linear leaf and glabrous bracteole in *S.graminea*. Furthermore, the capsule of *S.aprica* has 4 seeds vs. 8 seeds in *S.graminea*.

*Hance 536* from CAL [CAL0000019794] was designated as the lectotype of *Gutzlaffiaaprica* by Albertson and Wood (2012). On examination, we found a duplicate of this collection deposited at GH [GH00387581]. The specimen has *Hance*’s handwriting as follows: *Gutzlaffiaaprica* Hance. This specimen has branches, leaves and inflorescences, and it is the best preserved.

The original protologue of *Gutzlaffiapedunculata* was based on *Kerr 1430* (Craib 1911). We found that this collection has six duplicates. Two sheets are deposited at K [K001514863, K001514864] and one is housed at each of BM [BM000796839], C [C10005192], L [L2832219] and P [P00719397]. All duplicates are in good shape. We select K001514863 as the lectotype because it has more mature leaves, inflorescences and flowers.

#### ﻿2. Strobilanthes articulata

Taxon classificationPlantaeLamialesAcanthaceae

J.B.Imlay, Bull. Misc. Inform. Kew 1939(3): 121. 1939

4196D77F-0A9A-513E-ADE1-38D80D604430

Fig. 10A

##### Type.

Thailand, **Chanthaburi**, Khao [Kao] Soi Dao, 12 Dec 1924, *Kerr 9630* (lectotype BM [BM001191001!] designated here; isolectotypes ABD [ABDUH:2/887 image!], BK [257638!], C [C10005193 image!], K [K001096856!, K001096857!], KYO!).

##### Distribution.

Endemic to Thailand.

##### Ecology.

In evergreen forest, often on rocks; 1,300 m alt, flowering and fruiting December.

##### Specimens examined.

Thailand, South-eastern: **Chanthaburi**, Khao Soi Dao, 1,300 m alt., 12 Dec1924, *Kerr 9630* (BK, BM, K-2 sheets, KYO).

##### Preliminary conservation status assessment.

This species is only known from its type locality and is assessed as Data Deficient (DD) following IUCN (2022). More field work is needed to assess the conservation status of *S.articulata*.

##### Notes.

*Strobilanthesarticulata* superficially resembles *S.dimorphotricha* Hance in having zigzag stems in the upper parts, strongly unequal leaf pairs and glabrous and caducous bracts, but it differs in having no bracteoles vs. present in *S.dimorphotricha*.

*Strobilanthesarticulata* was described by Imlay (1939) based on *Kerr 9630* which has seven duplicates. Two of which are at K [K001096856, K001096857] and one at each of ABD [ABDUH:2/887], BK [257638], BM [BM001191001], C [C10005193] and KYO. BM001191001 has *Imlay*’s handwriting as follows: “*Strobilanthesarticulata* Imlay Type no.”, and it also has the mature fruit and corolla which correspond with the protologue. Therefore, we select it as the lectotype.

#### ﻿3. Strobilanthes brandisii

Taxon classificationPlantaeLamialesAcanthaceae

T.Anderson, J. Linn. Soc., Bot. 9: 475. 1867.

FE8DA3A4-C0C7-5847-8B6F-A2AA24C8A20B

Figs 1 , 10A

Strobilanthes evrardii var. parviflora J.B.Imlay, Bull. Misc. Inform. Kew 1939(3): 119. 1939. Type: Thailand, Ranong, Kraburi, Klong [Klawng] Wa, 50 m alt., 24 Dec 1928, *Kerr 16335* (lectotype BM [BM000906338!] designated here; isolectotypes ABD [ABDUH:2/906 image!], K [K001514907!]), syn. nov.

##### Type.

Myanmar, Hills of E. Tonghoo, without date, *Brandis 824* (lectotype CAL [CAL0000019781 image!] designated by Albertson and Wood 2012, pg. 54).

**Figure 1.**
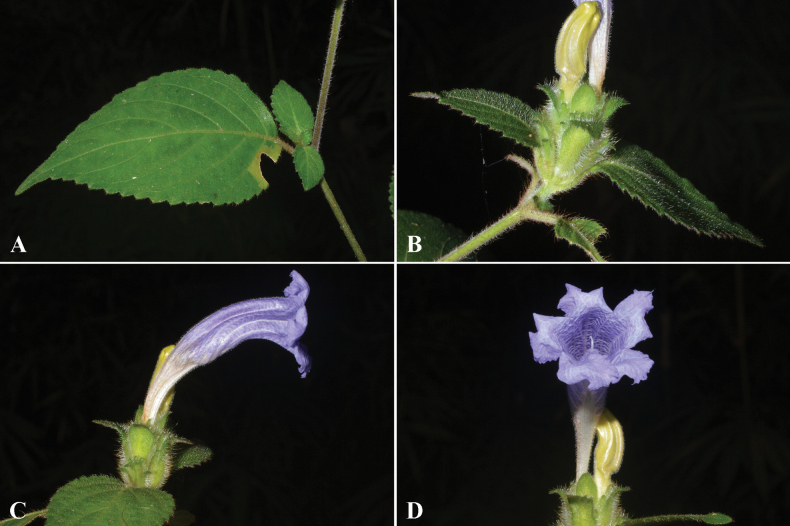
*Strobilanthesbrandisii* T.Anderson **A** stem and leaves **B** inflorescences **C** corolla, side view **D** corolla and stigma.

##### Distribution.

Myanmar, Thailand, Laos.

##### Ecology.

In partly shaded places of evergreen forest or mixed deciduous with bamboo; 10–1,531 m alt, flowering and fruiting from October to May.

##### Selected specimens examined.

Thailand, Northern: **Mae Hong Son**, Mae Tala, 1,376 m alt., 28 Apr 2014, *Norsaengsri 10909* (QBG); **Chiang Mai**, Doi Suthep-Pui NP, ca. 920 m alt., 19 May 1912, *Kerr 2604* (BM, E); ibid., Phrao, 1,050 m alt., 5 Dec 1990, *Hansen 44621* (CMU); **Tak**, Mae Sot, Khao Phra War, 695–800 m alt., 21 Jan 1983, *Koyama* et al. *32828* (BKF, K, KYO-2 sheets, L); **Phitsanulok**, Phu Hin Rong Kla NP, 1,300 m alt., 14 Oct 1998, *Suksathan 1306* (QBG); North-eastern: **Loei**, Phu Kradueng NP, 1,150–1,250 m alt., 1 Nov 1984, *Murata* et al. *42628* (BKF, L); ibid., Phu Luang WS, 980–1,531 m alt., 19 Feb 1983, *Koyama* et al. *33679* (BKF, L), *33686* (BKF, L); ibid., 19 Nov 2019, *Kladwong 503* (KKU); Eastern: **Nakhon Ratchasima**, Khao Yai NP, 1,170 m alt., 9 Oct 1979, *Shimizu* et al. *18097* (BKF, K, KYO, L); South-western: **Kanchanaburi**, Si Sawat, Tham Than Lod NP, Khao Kamphaeng, 1,100–1,370 m alt., 30 Nov 1982, *Koyama* et al. *30481* (BKF, KYO); South-eastern: **Chanthaburi**, Khao Soi Dao, 1,100–1,400 m alt., 12 Dec 1924, *Kerr 9629* (BK, BM, K); **Trat**, Khao [Kao] Kuap, 800 m alt., 24 Dec 1919, *Kerr 17792* (BK, BM, K); Peninsular: **Chumphon**, Ban Thung [Tung] Maha, 10 m alt., 10 Jan 1927, *Kerr 11363* (BK, BM); ibid., Lang Suan, 800 m alt., 22 Feb 1927, *Kerr 12074* (BK, K); **Ranong**, Kraburi, Klong [Klawng] Wa, 50 m alt., 24 Dec 1928, *Kerr 16335* (ABD, BM, K).

##### Preliminary conservation status assessment.

This species has an Extent of Occurrence (EOO) of 303,322.469 km^2^ and an Area of Occupancy (AOO) of 56.000 km^2^ and is assessed as Least Concern (LC) following IUCN (2022).

##### Notes.

*Strobilanthesbrandisii* is similar to *S.consors* C.B.Clarke in having decumbent habit, white sericeous obovate to oblanceolate or spathulate bracts and curved corolla. It differs in having an acute apex to the calyx lobes and linear bracts. Moreover, the bracts of *S.brandisii* are flat vs. curved in *S.consors*.

*Kerr 16335* was mentioned as the type of S.evrardiivar.parviflora (Imlay 1939). The type has three duplicates, one at each of ABD [ABDUH:2/906], BM [BM000906338] and K [K001514907]. BM000906338 has *Imlay*’s handwriting as follows “StrobilanthesevrardiiR. Ben.var.parviflora J.B.Imlay Type no. of var.”. Additionally, this specimen has a flower and the corolla length that agrees with the protologue. Therefore, the sheet BM000906338 is selected as the lectotype.

We examined the type of S.evrardiivar.parviflora and found it conspecific with *S.brandisii*.

#### ﻿4. Strobilanthes capitata

Taxon classificationPlantaeLamialesAcanthaceae

(Nees) T.Anderson, J. Linn. Soc., Bot. 9: 475. 1867.

8867436D-962C-5662-A184-358D374CAB39

Fig. 10B

Goldfussia capitata Nees, Pl. Asiat. Rar. 3: 88. 1832. Type: Nepal, 1821, *Wall. Numer. List: 2351*, 1831–1832 (lectotype K-W [K001115708!] designated here; isolectotypes BM [BM000793162!], GZU [GZU000251594 image!, GZU000251595 image!], K [K000883084!]).
Ruellia capitata Wall., Numer. List [Wallich] n. 2351. 1830, nom. nud.

##### Type.

Based on *Goldfussiacapitata* Nees

##### Distribution.

India, Bhutan, Nepal, Myanmar, China, Thailand.

##### Ecology.

In mixed deciduous forest or evergreen forest near waterfall; 237–2,190 m alt., flowering and fruiting from September to March.

##### Selected specimens examined.

Thailand, Northern, **Mae Hong Son**, Khun Yuam, Huai Yuak village, 500 m alt., 13 Jan 1983, *Koyama* et al. *32434* (KYO, L); ibid., Mueang, Doi Mae Sakut, 800–1,000 m alt., 23 Sept 1995, *Nanakorn* et al. *4654* (QBG-2 sheets); ibid., Pang Mapha, Tham Lot, 850 m alt., 10 Nov 2004, *Maxwell 04-682* (BKF, CMUB, L-3 sheets); **Chiang Mai**, Mae Chaem, Huai Hom, Ban Wat Chan, 1,000 m alt., 2 Dec 2007, *Srisanga* et al. *3121* (KYO, QBG); **Lamphun**, Doi Khun Tan NP, 925 m alt., 29 Jan 1994, *Maxwell 94-135* (BKF, CMUB); **Phrae**, Song, Mae Tom NP, 400 m alt., 14 Dec 1993, *Maxwell 93-1499* (CMUB, L-2 sheets); **Tak**, Mae Sot, Khao Phra War, 700–850 m alt., 12 Oct 1979, *Shimizu* et al. *18428* (BKF, K, KYO, L); North-eastern: **Loei**, Phu Luang WS, 1,300–1,562 m alt., 5 Dec 1965, *Tagawa* et al. *1605* (BKF, KYO-2 sheets, L).

##### Preliminary conservation status assessment.

This species has an Extent of Occurrence (EOO) of 63,122.391 km^2^ and an Area of Occupancy (AOO) of 52.000 km^2^ and is assessed as Least Concern (LC) following IUCN (2022).

##### Notes.

*Strobilanthescapitata* resembles *S.kerrii* Craib and *S.speciosa* Blume in having a straight corolla and nodding short stamens. The species differs from the former as it lacks purplish hairs on the stems, petioles and peduncles and from the latter by having ovate or oblong-elliptic and curved bracts and blue to purplish-blue flowers.

Nees (1832) described *Goldfussiacapitata* based on *Wallich 2351* which has five duplicates, one at each of BM [BM000793162], K [K000883084] and K-W [K001115708] and two at GZU [GZU000251594, GZU000251595]. The sheet K001115708 is the best preserved and has completely mature leaves, inflorescences and flowers. Therefore, we select it as the lectotype.

#### ﻿5. Strobilanthes chiangdaoensis

Taxon classificationPlantaeLamialesAcanthaceae

Terao, Acta Phytotax. Geobot. 32(1–4): 31. 1981.

A37E43CB-B17A-5905-8EF2-D81B2F447D99

Figs 2 , 10B

##### Type.

Thailand, Chiang Mai, Doi Chiang Dao, 4 Dec 1965, *Hennipman 3187* (holotype L [L0002847!]; isotypes C [C10005196 image!], BKF!, K [K001514861!], KYO!).

**Figure 2.**
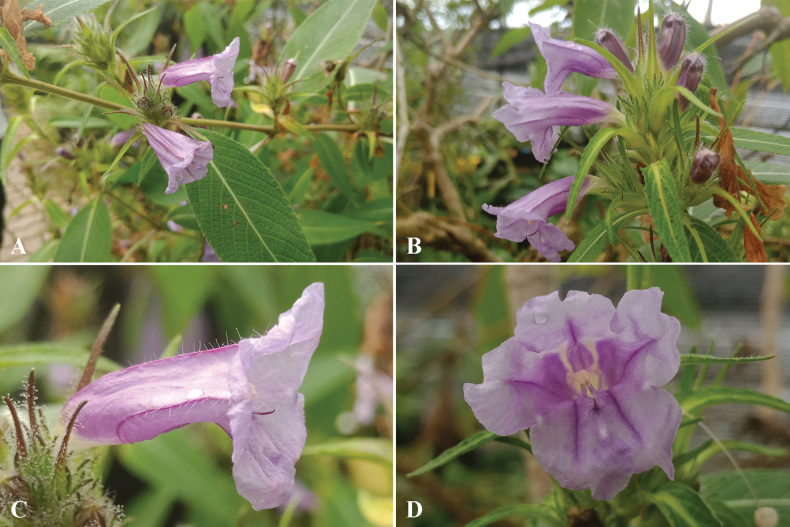
*Strobilantheschiangdaoensis* Terao **A** stem and leaves **B** inflorescences **C** corolla, side view **D** corolla lobes, anthers and stigma. Photos taken from cultivated plant from QBG nursery.

##### Distribution.

Endemic to Thailand.

##### Ecology.

On rugged limestone ridge, open areas in mixed evergreen and deciduous forests; 800–2,190 m alt., m alt., flowering and fruiting from September to March.

##### Selected specimens examined.

Thailand, Northern: **Mae Hong Son**, Pang Mapha, Tham Lot, 925 m alt., 11 Nov 2004, *Maxwell 04-698* (BKF, CMUB, L2 sheets); **Chiang Mai**, Doi Chiang Dao, 1050 m alt., 4 Dec 1965, *Hennipman 3187* (C, BKF, K, KYO, L); ibid., 500–1600 m alt., 3 Jan 1966, *Tagawa* et al. *4039* (AAU, BKF, K, KYO-3 sheets, P); ibid., 1,510–2,190 m alt. 8 Feb 1983, *Koyama* et al. *33225* (BKF, KYO); **Chiang Rai**, Mae Fa Luang, Doi Tung, 1,300 m alt., 22 Oct 1995, *Pooma 1176* (BKF, CMUB); ibid., 1,400 m alt., 5 Nov 2004, *Maxwell 04-573* (L); ibid., Huai Khrai, near Wat Phra That Doi Tung, 1,359 m alt., 15 Sept 2012, *Chamchumroon* et al. *VC 5434* (BKF-2 sheets, E); **Phayao**, Chiang Kham, Doi Pha Dam, Ban Pang Tham, 1,030 m alt., 14 Nov 2012, *La-ongsri* et al. *25886* (QBG); Wang Nuea, Ban Paak Bok, 1,100 m alt., 19 Jan 2006, *Suksathan 3659* (QBG); **Lampang**, Ngao, Ban Pha Daeng, 800 m alt., 16 Jan 2006, *Suksathan 3614* (KYO, QBG).

##### Preliminary conservation status assessment.

This species has an Extent of Occurrence (EOO) of 19,556.802 km^2^ and an Area of Occupancy (AOO) of 32.000 km^2^ and is assessed as Vulnerable (VU), B1 a, b (i, ii, iii) following IUCN (2022). This species grows among rugged limestone rocks which occur at the top of limestone mountain. This habitat is subject to increasing droughts and fires leading to decline of *S.chiangdaoensis*.

##### Notes.

*Strobilantheschiangdaoensis* resembles *S.esquirolii* in having oblong-lanceolate bracts and bracteoles. However, the bracts and bracteoles of *S.chiangdaoensis* are glabrous or sparsely hairy on the adaxial surface and pubescent on the abaxial surface vs. sericeous on both surfaces in *S.esquirolii*. Moreover, the capsules of *S.chiangdaoensis* have two seeds with two lower rudimentary ovules vs. four seeds without rudimentary ovules in *S.esquirolii*.

#### ﻿6. Strobilanthes consors

Taxon classificationPlantaeLamialesAcanthaceae

C.B.Clarke, Bot. Jahrb. Syst. 41(2): 66. 1907.

1E7EF529-40B3-5C9E-8487-FE9AD84676EF

Fig. 10B

##### Type.

Thailand, Chiang Mai, Doi Suthep, 13 Dec 1904, *Hosseus 256* (lectotype M [M0168698 image!] designated here; isolectotypes B [B101185735 image!], BM [BM000906339!], E [E00273462!], K [K001514905!], L [L2841550!], P [P00719278 image!]).

##### Distribution.

Chamchumroon, Myanmar, Thailand.

##### Ecology.

In evergreen forest, granite bedrock; 250–2,500 m alt., flowering and fruiting from September to May.

##### Selected specimens examined.

Thailand, Northern: **Mae Hong Son**, Mae Tala, 1,376 m alt., 28 Apr 2014, *Norsaengsri 10909* (QBG); **Chiang Mai**, Doi Inthanon NP, Doi Ang Ka, 1,600 malt., 26 Dec 1935, *Garrett 1026* (BKF, K-2 sheets, KYO); ibid., Fang, Doi Pha Hom Pok NP, 1,900–2,000 m alt., 11 May 1905, *Hosseus 606* (M); ibid., Doi Suthep-Pui NP, 13 Dec 1904, *Hosseus 256* (B, BM, E, K, L, M, P); ibid., ca. 1,680 m alt., 10 Jan 1911, *Kerr 2279* (BM, K-2 sheets, L, TCD); ibid., ca. 1,520 m alt., 24 Dec 1911, *Kerr 2279A* (BM-2 sheets, E, L, TCD); ibid., ca. 1,580 m alt., 22 Sept 1912, *Kerr 2715* (AAU, BM-2 sheets, E, K); ibid., 1,510 m alt., 20 Dec 2009, *Balslev* et al. *10029* (AAU); **Nan**, Bo Kluea, Sapan Waterfall, 600 m alt., 17 Nov 1993, *Larsen* et al. *44464* (AAU); ibid., 780 m alt., 2 Sept 2000, *Srisanga 1587* (BKF, QBG, CMUB); **Chiang Rai**, Doi Chang, 1,260–1,765 m alt., 11 Jan 1922, *Rock 1771* (E, US); **Lamphun**, Mae Tha, Doi Khun Tan NP, 1,200 m alt., 20 Nov 1993, *Maxwell 93-1407* (BKF-2 sheets, CMUB, L); **Lampang**, Chae Son NP, 875 m alt., 24 Oct 1995, *Maxwell 95-991* (BKF, CMUB); ibid., Doi Luang NP, 1,125 m alt., 8 Nov 1998, *Petrmitr 335* (CMUB, L); North-eastern: **Loei**, Phu Kradueng NP, 1,250 m alt., 9 Sept 1988, *Takahashi & Tamura 63463* (BKF); ibid., Phu Luang WS, 1,150–1,530 m alt., 24 Dec 1982, *Koyama* et al. *31617* (KYO-2 sheets); ibid., Phu Ruea NP, 980–1,151 m alt., 23 Dec 1982, *Koyama* et al. *31543* (BKF, KYO).

##### Preliminary conservation status assessment.

This species has an Extent of Occurrence (EOO) of 73,029.391 km^2^ and an Area of Occupancy (AOO) of 76.000 km^2^ and is assessed as Least Concern (LC) following IUCN (2022).

##### Notes.

*Hosseus 256* and *Hosseus 606* were cited in the original protologue of *S.consors* (Hosseus 1907). *Hosseus 256* has seven duplicates one in each of B [B101185735], BM [BM000906339], E [E00273462], K [K001514905], L [L2841550], M [M0168698] and P [P00719278] whereas *Hosseus 606* has only one duplicate deposited at M [M0168699]. M0168698 has the original label “*Strobilanthesconsors* sp.nova.” and the original description in *Clarke*’s handwriting. Moreover, this specimen has mature leaves, inflorescences and flowers. We, therefore, select the sheet M0168698 as the lectotype of *S.consors*.

#### ﻿7. Strobilanthes cruciata

Taxon classificationPlantaeLamialesAcanthaceae

(Bremek.) Terao, Acta Phytotax. Geobot. 31(1–3): 59. 1980.

03580FD1-38D2-57BF-B8DC-A3443BFCBAE5

Fig. 10C

Tetragoga cruciata Bremk., Verh. Kon. Ned. Akad. Wetensch., Afd. Natuurk., Sect. 2. 41(1): 300. 1944. Type: Indonesia, Sumatra, 1 May 1918, *Lörzing 5668* (holotype L [L0002848!]; isotypes BO [BO1352476 image!, BO1352477 image!], GH [GH00295522 image!], SING [SING0045507 image!]).
Tetragoga nagaensis Bremek., Verh. Kon. Ned. Akad. Wetensch., Afd. Natuurk., Sect. 2. 41(1): 299. 1944. Type: India, Nagaland, Dec 1907, *Meebold 4891* (holotype B [B100002761 image!]).

##### Type.

Based on *Tetragogacruciata* Bremk.

##### Distribution.

India, Myanmar, China, Thailand, Vietnam, Indonesia.

##### Ecology.

In hilly evergreen forest; 150–1,700 m alt, flowering and fruiting from July to May.

##### Selected specimens examined.

Thailand, Northern: **Nan**, Doi Phu Kha NP, 1,700 m alt., 28 July 1992, *Larsen* et al. *43704* (AAU, P); ibid., 1,700 m alt., 26 May 2000, *Srisanga 1445* (QBG); ibid., 1,680 m alt., 11 Nov 2000, *Srisanga 1758* (BKF- 2 sheets, QBG); ibid., Pua, 1,650 m alt., 10 May 2006, *Srisanga 2762* (CMUB, KYO, QBG); Peninsular: **Chumphon**, Marine Nature Study Center, 9 Apr 2008, *Wessumritt 113* (QBG); ibid., Phato, Ban Racha Krude, 150–200 m alt., 6 July 1992, *Larsen 43165* (AAU, BKF, P).

##### Preliminary conservation status assessment.

This species has an Extent of Occurrence (EOO) of 2,663,189.074 km^2^ and an Area of Occupancy (AOO) of 44.000 km^2^ and is assessed as Least Concern (LC) following IUCN (2022).

##### Notes.

*Strobilanthescruciata* resembles *S.falconeri* T.Anderson in having leaf-like bracts with a petiolar base, but it is distinguishable from *S.falconeri* T.Anderson by its white corolla and glabrous ovary.

#### ﻿8. Strobilanthes dimorphotricha subsp. rex

Taxon classificationPlantaeLamialesAcanthaceae

(C.B.Clarke) J.R.I.Wood, Kew Bull. 61(1): 2006.

28D40B93-CAC9-5C6C-8E46-E34E3BD3CF69

Figs 3 , 10C

Strobilanthes anfractuosa C.B.Clarke, Bot. Jahrb. Syst. 41(2): 66. 1907. Type: Thailand, Doi Inthanon [Doi Angka], 1150 m., 17 Jan 1905, *Hosseus 336* (holotype M [M0168700 image!]; isotypes BM!, K [K001514926!], P [P00719248 image!]).
Goldfussia anfractuosa (C.B.Clarke) Bremek., Verh. Kon. Ned. Akad. Wetensch., Afd. Natuurk., Sect. 2. 41(1): 269. 1944. Type: Based on Strobilanthesanfractuosa C.B.Clarke
Strobilanthes pentastemonoides (Nees) T.Anderson var. anfractuosa (C.B.Clarke) Benoist in Lecomte *et al*., Fl. Indo-Chine 4: 667. 1935. Type: Based on Strobilanthesanfractuosa C.B.Clarke
Strobilanthes rex C.B.Clarke, Bot. Jahrb. Syst. 41(2): 68. 1907. Type: Thailand, Doi Inthanon [Doi Anga], *Hosseus 352* (holotype M [M0168691]; isotypes BM [BM000793208!], C [C10005214 image!], CORD [CORD00005092 image!], E [E00749032!, E00749033!, E00749034!], K [K001514927!], P [P00719419 image!, P00719420 image!, P00719421 image!]).
Goldfussia rex (C.B.Clarke) Bremek., Verh. Kon. Ned. Akad. Wetensch., Afd. Natuurk., Sect. 2. 41(1): 283. 1944. Type: Based on Strobilanthesrex C.B.Clarke
Strobilanthes pentastemonoides (Nees) T.Anderson var. rex (C.B.Clarke) Benoist in Lecomte et al., Fl. Indo-Chine 4: 667. 1935. Type: Based on Strobilanthesrex C.B.Clarke

##### Type.

Based on *Strobilanthesrex* C.B.Clarke

**Figure 3.**
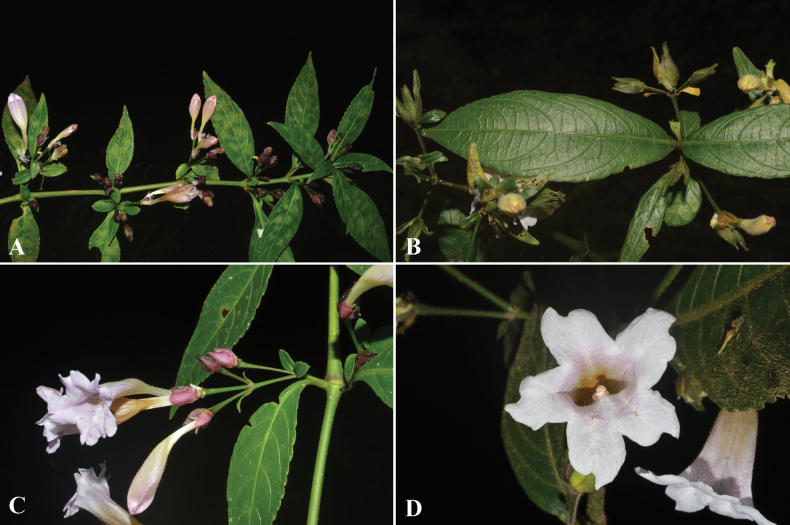
Strobilanthesdimorphotrichasubsp.rex (C.B.Clarke) J.R.I.Wood **A, B** stem, leaves and inflorescences **C** inflorescence **D** corolla lobes and anthers.

##### Distribution.

Myanmar, China, Thailand, Laos.

##### Ecology.

Common in evergreen forest; 375–2,540 m alt., flowering and fruiting from July to April.

##### Selected specimens examined.

Thailand, Northern: **Mae Hong Son**, Pai, Doi Kiew Lom, 1,490 m alt., 16 Jan 1983, *Koyama* et al. *32588* (BKF); **Chiang Mai**, Doi Chiang Dao WS, 13 Feb 1958, *Bunchuai 716* (BKF); ibid., 1,200 m alt., 17 Feb 1958, *Sørensen* et al. *1298* (E); ibid., 1,200–1,600 m alt., 6 Jan 1975, *Geesink* et al. *8117* (BKF, K, L); ibid., 14 Oct 2019, *Kladwong 489* (KKU); ibid., Doi Inthanon NP, ca. 2,500 m alt., 19 Jan 1905, *Hosseus 352* (BM, CORD, E-3 sheets, K, M, P-3 sheets); ibid., 17 Jan 1905, *Hosseus 334* (BM); ibid., 17 Jan 1905, *Hosseus 336* (BM, K, M, P); ibid., Doi Pha Hom Pok NP, 1,600–2,350 m alt., 12 Feb 1983, *Koyama* et al. *33423* (BKF); ibid., Doi Suthep-Pui NP, 14 Dec 1904, *Hosseus 244* (BM, E, K, L); ibid., 900 m alt., 16 Jan 1910, *Kerr 935* (AAU, BM, L); ibid., 1,676 m alt., 20 Nov 1910, *Kerr 1548* (BM); ibid., 900 m alt., 8 Jan 1911, *Kerr 1568A* (BM-2 sheets, K, TCD); ibid., 1,676 m alt., 10 Dec 1911, *Kerr 1568B* (AAU, BM, K, L); ibid., Doi Pui Campground trail, 1,510 m alt., 20 Dec 2019, *Balslev* et al. *10024* (AAU); ibid., 4 Oct 2019, *Kladwong 473* (KKU); **Chiang Rai**, Doi Luang NP, Pu Kaeng Waterfall, 592 m alt., 2 Mar 2015, *Norsaengsri 11746* (QBG); ibid., Mae Sai, 1,350 m alt., 4 Feb 2006, *Maxwell 06-129* (QBG, CMUB, L); ibid., Tham Luang-Khun Nam Nang Norn NP, 800 m alt., 22 Jan 2000, *Suksathan 2270* (QBG); **Phayao**, Chiang Kham, Phu Lang Ka, 1,500 m alt., 18 Jan 2006, *Suksathan 3641* (QBG, CMUB); Nan, Doi Phu Kha NP, 1,500–1,600 m alt., 13 Dec 1990, *Larsen* et al. *41910* (AAU); ibid., 1,700 m alt., 28 July 1992, *Larsen* et al. *43702* (AAU); **Lampang**, Chae Son NP, 1,150 m alt., 7 Jan 1996, *Maxwell 96-18* (BKF, CMUB); **Uttaradit**, Phu Soi Dao NP, Sai Thong Waterfall, 1,615 m alt., 17 Nov 2009, *Norsaengsri & Intamusik 6162* (QBG); **Tak**, Mae Sot, Pha Charoen Waterfall, 680 m alt., 10 Feb 2002, *Simpson* et al. *2078* (K, TCD); **Phitsanulok**, Chat Trakan, 22 Jan 2009, *Maknoi 3002* (QBG); ibid., Phu Hin Rong Kla NP, 1,400–1,600 m alt., 10–11 Dec 1990, *Larsen* et al. *41827* (AAU) & *41878* (AAU); North-eastern: **Phetchabun**, Nam Nao NP, 18 Jan 2003, *Chantaranothai* et al. *s.n.* (BKF); **Loei**, Phu Suan Sai NP, 19 Dec 2006, *Maknoi* et al. *1238* (QBG-2 sheets); ibid., Phu Kradueng NP, 1,100–1,200 m alt., 28 Nov 1965, *Tagawa 491* (BKF, KYO-2 sheets); ibid., Wang Kwang Waterfall, 1,190–1,250 m alt., 16 Nov 1979, *Shimizu* et al. *23219* (BKF, KYO, L); Eastern: **Chiyaphum**, Nam Phrom, 600 m alt., 10 Dec 1971, *van Beusekom* et al. *4097* (BKF, K, L); South-western: **Kanchanaburi**, Ta Kanun, 400 m alt., 19 Jan 1962, *Kerr 10267* (BK, BM, K); Peninsular: **Ranong**, Kaper, Khao Pawta Luang Kaeo, 940–1,300 m alt., 10 Dec 1979, *Shimizu* et al. *26724* (BKF, L), *26739* (BKF), *26841* (BKF) & *26887* (BKF, L); **Nakhon Si Thammarat**, Lan Saka, Khao Luang, 19 Nov 1955, *Snan 312* (BKF).

##### Preliminary conservation status assessment.

This species has an Extent of Occurrence (EOO) of 295,960.413 km^2^ and an Area of Occupancy (AOO) of 176.000 km^2^ and is assessed as Least Concern (LC) following IUCN (2022).

##### Notes.

Strobilanthesdimorphotrichasubsp.rex differs consistently from subsp. dimorphotricha Hance from China and Vietnam in the rigid and subequal or sometimes unequal leaves. Subsp. rex appears similar to *S.paniculiformis* J.R.I.Wood in which the calyx lobe has 1 lobe longer than others. However, it differs by having ovate or elliptic-lanceolate bracts vs. ovate-orbicular in *S.paniculiformis*.

#### ﻿9. Strobilanthes echinata

Taxon classificationPlantaeLamialesAcanthaceae

Nees in Wall., Pl. Asiat. Rar. 3: 85. 1832.

571B9CA5-7E4A-51E6-A096-79CDC751FEAC

Fig. 10C

Goldfussia echinata (Nees) N.P.Balakr., Fl. Jowai 2: 355. 1983. Type: Based on Strobilanthesechinata Nees
Dicliptera crenata Miq., Fl. Ned. Ind. 2: 844. 1858. Type: Indonesia, Sumatra, *Horsfield s.n.* (isotype BM [BM000884896!]).
Strobilanthes jugorum Benoist, Bull. Soc. Bot. France 81: 601. 1934. Type: Vietnam, Tonkin, Chapa, Sept 1929, *Pételot s.n.* (holotype P [P00204976 image!]).
Tetraglochidium jugorum (Benoist) Bremek., Dansk Bot. Ark. 23: 207. 1965. Type: Based on Strobilanthesjugorum Benoist
Strobilanthes latibracteata J.B.Imlay, Bull. Misc. Inform. Kew 1939(3): 122. 1939. Type: Thailand, Trang, Khao [Kao] Soi Dao, 28 Apr 1930, *Kerr 19190* (lectotype BM [BM000793157!] designated here; isolectotypes ABD [ABDUH:2/908 image!], BK [257645!]).
Strobilanthes maingayi C.B.Clarke in Hook.f., Fl. Brit. India 4(11): 448. 1884. Type: Malaya, *Maingay 1182* (lectotype K [K001514853!] first step designated by Bennett et al. (2008), second step designated here; isolectotypes BM [BM00088495!], K [K001514854!]).
Tetraglochidium maingayi (C.B.Clarke) Bremek., Verh. Kon. Ned. Akad. Wetensch., Afd. Natuurk., Sect. 2. 41(1): 221. 1944. Type: Based on Strobilanthesmaingayi C.B.Clarke
Tetraglochidium maingayi var. paucinervium Bremek., Dansk Bot. Ark. 23: 206. 1965. Type: Thailand, Prachinburi, Khao Khieo, 20 June 1963, *Larsen 10159* (holotype U [U0000138 image!]; isotypes AAU!, BKF [BKF080038!], C [C10005203 image!]).
Strobilanthes maingayi var. glabra [glaber]J.B.Imlay, Bull. Misc. Inform. Kew 1939(3): 119. 1939. Type: Thailand, Prachuap Khiri Khan, Khao [Kao] Luang, 5 July 1926, *Kerr 10835* (lectotype BM [BM000793159!] designated here; isolectotypes ABD [ABDUH:2/909 image!], BK [257644!], K [K001514916!, K001514917!]).
Strobilanthes pectinata var. acuminata J.B.Imlay in Kew Bull. 1939: 119. 1939. Type: Thailand, Khao [Kao] Luang, 29 Apr 1928, *Kerr 15464* (lectotype BM [BM000793158!] designated here; isolectotypes ABD [ABDUH:2/912 image!], BK [231580!], K [K001514913!, K001514914!]).
Strobilanthes echinata var. acuminata (J.B.Imlay) Bremek., Verh. Kon. Ned. Akad. Wetensch., Afd. Natuurk., Sect. 2. 41(1): 274. 1944. Type: Based on Strobilanthespectinatavar.acuminata J.B.Imlay.
Strobilanthes pectinata var. glandulosa J.B.Imlay, Bull. Misc. Inform. Kew 1939(3): 119. 1939. Type: Thailand, Nan, Pua, Mt. Pu Huat, 3 Mar 1921, *Kerr 4993* (lectotype BM [BM000793160!] designated here; isolectotypes ABD [ABDUH:2/910 image!], BK [231583!], K [K001514918!]).
Strobilanthes echinata var. glandulosa (J.B.Imlay) Bremek., Verh. Kon. Ned. Akad. Wetensch., Afd. Natuurk., Sect. 2. 41(1): 274. 1944. Type: Based on Strobilanthespectinatavar.glandulosa J.B.Imlay
Strobilanthes pectinata var. punctata J.B.Imlay, Bull. Misc. Inform. Kew 1939(3): 119. 1939. Type: Thailand, Satun, Khao [Kao] Keo Range, 12 Mar 1928, *Kerr 14512* (lectotype BM [BM000793156!] designated here; isolectotypes ABD [ABDUH:2/911 image!], BK [231579!]).
Strobilanthes echinata var. punctata (J.B.Imlay) Bremek., Verh. Kon. Ned. Akad. Wetensch., Afd. Natuurk., Sect. 2. 41(1): 274. 1944. Type: Based on Strobilanthespectinatavar.punctata J.B.Imlay
Ruellia pectinata Wall., Numer. List [Wallich] n. 2356. 1830, nom. nud.

##### Type.

India, Meghalaya, *de Silva* in *Wall. Numer. List: 2356*, 1831–1832 (lectotype K-W [K001115724!], designated by Bennett et al. 2008, pg. 133; isolectotype BM [BM000884989!]).

##### Distribution.

India, Bhutan, Myanmar, China, Thailand, Laos, Vietnam Cambodia, Malaysia, Indonesia.

##### Ecology.

In evergreen forest, granite bedrock; 310–2,580 m alt., flowering and fruiting from August to July.

##### Selected specimens examined.

Thailand, Northern: **Chiang Mai**, Doi Inthanon NP, 2,200–2,440 m alt., 2 May 1921, *Kerr 5308* (BK, BM, K); ibid., 2,170 m alt., 4 June 1930, *Garrett 564* (BKF, K-2 sheets, L); ibid., 1,200–2,580 m alt., 24 June 1978, *Phengklai* et al. *4066* (BKF, K, L, PSU); ibid., 2,565 m alt., 31 Dec 1989, *Maxwell 89-1629* (CMU, E, L); ibid., Fang, Doi Pha Hom Pok NP, 1,300 m alt., 12 Sept 1967, *Iwatsuki* et al. *9572* (BKF, K, KYO, L); **Nan**, Doi Phu Kha NP, 1,450 m alt., 25 June 1999, *Srisanga 735* (AAU, BKF, QBG, CMUB); ibid., Pua, Phu Huat, 1,500–1,600 m alt., 3 Mar 1921, *Kerr 4993* (ABD, BK, BM, K); **Uttaradit**, Phu Soi Dao NP, 1,960 m alt., 30 June 2009, *Intamusik* et al. *245* (QBG); North-eastern: **Loei**, Phu Luang WS, 1,200 m alt., 28 Aug 1996, *Phengklai & Fukuoka 10096* (BKF-3 sheets, QBG-2 sheets); Eastern: **Chaiyaphum**, Thung Kamang, 850 m alt., 1 June 1974, *Geesink* et al. *7129* (AAU, BKF, K, L); **Nakhon Ratchasima**, Khao Yai NP, 600–800 m alt., 7 July 1963, *Kasem 288* (BK); ibid., Khao Khieo, 1,300 m alt., 29 Aug 1963, *Smitinand & Sleumer s.n.* (BKF); South-western: **Prachuap Khiri Khan**, Khao Luang, 1,000 m alt., 5 July 1926, *Kerr 10835* (ABD, BK, BM, K-2 sheets); Central: **Saraburi**, Khao Khieo, 1,000 m alt., 8 June 1979, *Vidal* et al. *6360* (AAU, BKF, K, KYO, L, P); South-eastern: **Prachinburi**, Khao Khieo, 1,300 m alt., 20 June 1963, *Larsen 10159* (AAU, BKF, C, U); **Rayong**, Khao Cha Moa-Khao Wong NP, 650 m alt., 24 Nov 1979, *Shimizu* et al. *23465* (KYO); **Chanthaburi**, Khao Khitchakut NP, Krating Waterfall, 310 m alt., 29 Nov 1979, *Shimizu* et al. *23940* (BKF, KYO-2 sheets, L); **Trat**, Khao Kuap, 22 May 1930, *Put 2940* (BK, BM, K); Peninsular: **Ranong**, Kaper, Khao Pawta Luang Kaeo, 940–1200 m alt., 9 Dec 1979, *Shimizu* et al. *26595* (BKF, KYO); **Krabi**, Khao Phanom Bencha NP, 1,350 m alt., 8 Jan 2006, *Gardner ST2182* (BKF-2 sheets, K-2 sheets); **Nakhon Si Thammarat**, Khao Luang, 900 m alt., 29 Apr 1928, *Kerr 15464* (ABD, BK, BM, K-2 sheets); ibid., 3 May 1941, *Smitinand 827* (BKF, L); ibid., 1,200–1,300 m alt., 24 Feb 1995, *Larsen* et al. *45973* (AAU); **Trang**, Palian, Khao Soi Dao, 800 m alt., 28 Apr 1930, *Kerr 19190* (ABD, BK, BM); ibid., Yan Ta Khao, Khao Banthat, summit area of Phu Pha Mek, 1,240 m alt., 7 Apr 2003, *Middleton* et al. *1995* (BKF, E) & *2001* (BKF); **Satun**, Khao [Kao] Keo Range, 600 m alt., 12 Mar 1928, *Kerr 14512* (ABD, BK, BM); ibid., 700 m alt., 12 Mar 1928, *Kerr 14528* (BK, BM, K); **Songkhla**, Hat Yai, Ton Nga Chang Waterfall, 21 Aug 1992, *Niyomdham 3066* (BKF-2 sheets); **Pattani**, Khao [Kao] Kala Kiri, 800–900 m alt., 1 Apr 1928, *Kerr 14954* (BK, BM, K); **Yala**, Bannang Sata, Khao Pok Yok, 1,000 m alt., 10 Oct 1991, *Larsen* et al. *42276* (AAU, BKF).

##### Preliminary conservation status assessment.

This species has an Extent of Occurrence (EOO) of 469,202.533 km^2^ and an Area of Occupancy (AOO) of 88.000 km^2^ and is assessed as Least Concern (LC) following IUCN (2022).

##### Notes.

*Strobilanthesechinata* differs from *S.cruciata* in its dentate or fimbriate or dentate-crenate vs. acuminate on the apex bracteoles and calyx. In addition, the bracts of *S.echinata* are sessile vs. bract with petiolar base in *S.cruciata*.

*Strobilanthesmaingayi* was described based on *Maingay 1182* at K (Clarke 1884). Material at K was considered to be the holotype by Bennett et al. (2008). However, there is no indication in Clarke (1884) that only the material now in K was studied. We also found that there are three duplicates of *Maingay 1182*, two of which are deposited at K [K001514853, K001514854] and the other one at BM [BM00088495]. K001514853 has more leaves and inflorescences than the others. Bennett et al. (2008) can be considered as first step lectotypification (Turland 2019). We therefore, undertake the second step and select K001514853 as the lectotype.

*Kerr 19190* was cited in the protologue of *S.latibracteata* (Imlay 1939). This number has three duplicates each one was deposited at ABD [ABDUH:2/908], BK [257645] and BM [BM000793157]. We found that the sheet BM000793157 has *Imlay*’s handwriting labelled as “*Strobilantheslatibracteata* Imlay Type no.” and the specimen has well-preserved bracts as well as corolla. Furthermore, the size of the leaf, bract and corolla matches with the protologue. Therefore, the sheet BM000793157 is selected as the lectotype.

*Kerr 10835* was mentioned as the type of S.maingayivar.glabra (Imlay 1939). [The varietal name was originally published as ‘*glaber*: but as *Strobilanthes* is feminine we follow Bennett et al. 2008 and use *glabra*]. *Kerr 10835* has five duplicates, two of which are at K [K001514916, K001514917] and one each at ABD [ABDUH:2/909], BK [257644] and BM [BM000793159]. The sheet BM000793159 agreed with the protologue based on leaf size and has *Imlay*’s handwriting as follows: “StrobilanthesmaingayiC.B.Clarkevar.glabra Imlay Type of var.” Therefore, we designate the sheet BM000793159 as the lectotype.

*Kerr 15464* was cited in the protologue of S.pectinatavar.acuminata (Imlay 1939). This number has five duplicates, two of which were deposited at K [K001514913, K001514914] and each one was housed at ABD [ABDUH:2/912], BK [231580], BM [BM000793158]. The sheet BM000793158 has *Imlay*’s handwriting as follows: “StrobilanthespectinataT. Anders.var.acuminata Imlay Type of var.” and has well-preserved leaves. Therefore, the sheet BM000793158 is selected as the lectotype and the other duplicates are isolectotypes.

Imlay (1939) described a new taxon, S.pectinatavar.glandulosa based on *Kerr 4993* which has four duplicates one each at ABD [ABDUH:2/910], BK [231583], BM [BM000793160] and K [K001514918]. We select the sheet BM000793160 as the lectotype because it has glandular hairs on the stem which correspond with the protologue. Additionally, it also has *Imlay*’s handwriting as follows: “StrobilanthespectinataT. Anders.var.glandulosa Imlay”.

Var. pectinata was described as a new taxon by Imlay (1939) based on *Kerr 14512* which has three duplicates, each one was deposited in ABD [ABDUH:2/911], BK [231579] and BM [BM000793156]. The sheet BM000793156 has the size of bracts, calyx and fruits corresponding with the protologue and it also has *Imlay*’s handwriting as follows: “StrobilanthespectinataT. Anders.var.punctata Imlay Type no. of var.”. Therefore, the sheet BM000793156 is selected as the lectotype.

#### ﻿10. Strobilanthes erecta

Taxon classificationPlantaeLamialesAcanthaceae

C.B.Clarke, Bot. Jahrb. Syst. 41(2): 67. 1907.

DF6F18F0-8C2B-524B-93F4-C687351BC050

Fig. 10D

Goldfussia laotica Bremek., Proc. Kon. Ned. Akad. Wetensch. C 60: 3. 1957. Type: Laos, Xieng Khouang, Vidal 1685 (holotype U [U0000028!]; isotype P [P04366109 image!]).
Strobilanthes suborbicularis J.B.Imlay, Bull. Misc. Inform. Kew 1939(3): 118. 1939. Type: Thailand. Chiang Mai, Doi Inthanon [Doi Aang Ka], 2 Nov 1930, *Put 3302* (lectotype K n.v. designated by Son et al. (2018); isolectotypes ABD [ABDUH:2/914 image!], BK [257647!], BM [BM000906322!], C [C10005219 image!], KYO!).
Dossifluga suborbicularis (J.B.Imlay) Bremek., Verh. Kon. Ned. Akad. Wetensch., Afd. Natuurk., Sect. 2. 41(1): 235. 1944. Type based on Strobilanthessuborbicularis J.B.Imlay

##### Type.

Thailand, Doi Chiang Dao, 17 Feb 1905, *Hosseus 401a* (holotype M [M0168696 image!]; isotype P [P00719317 image!]).

##### Distribution.

Myanmar, China, Thailand, Laos, Vietnam.

##### Ecology.

In open hill evergreen scrub and pine forest on mountain top, sandy soil, limestone or granite bedrock; 980–2,200 m alt., flowering and fruiting from September to February.

##### Selected specimens examined.

Thailand, Northern: **Chiang Mai**, Doi Chiang Dao WS, 2,160 m alt., 17 Feb 1905, *Hosseus 401a* (M, P); ibid., 1,500–2,200 m alt., 3 Dec 1961, *Smitinand & Anderson 7305* (BKF-2 sheets); ibid., 2,000 m alt., 7 Dec 1965, *Hennipman 3273* (BKF, L); ibid., Chom Thong, Doi Inthanon NP, 2 Nov 1930, *Put 3302* (ABD, BM, C, K); ibid., 1,800 m alt., 18 Feb 1999, *Suksathan 1572* (QBG); ibid., 1,700 m alt., 22 Sept 2001, *Suksathan 3087* (QBG); ibid., 19 Nov 2020, *Kladwong 532 & 533* (KKU); **Nan**, Doi Phu Kha NP, Doi Phu Wae, 1,700 m alt., 10 Dec 1998, *Srisanga 412* (AAU, BKF, CMUB, KYO, QBG); North-eastern: **Loei**, Phu Luang WS, 1,300 m alt., 27 Nov 1959, *Bunpheng 955* (BKF); ibid., 1,500 m alt., 3 Jan 1983, *Niyomdham & Vidal 442* (AAU, BKF-2 sheets, P) & *501* (AAU, BKF-2 sheets, P); ibid., 1,400 m alt., 15 Apr 1968, *Chermsirivathana 872* (BK); ibid., 1,500 m alt., 26 Jan 1981, *Smitinand s.n.* (BKF); ibid., 19 Nov 2019, *Kladwong 501* (KKU).

##### Preliminary conservation status assessment.

This species has an Extent of Occurrence (EOO) of 312,285.154 km^2^ and an Area of Occupancy (AOO) of 44.000 km^2^ and is assessed as Least Concern (LC) following IUCN (2022).

##### Notes.

*Strobilantheserecta* resembles *S.phyllocephala* J.R.I.Wood & Scotland in the shape of its leaf base, but it differs in having ovate-elliptic or obovate and caducous bracts. Wood and Scotland (2006) treated *S.laotica* as a synonym of S.dimorphotrichasubsp.rex, but after investigation of type specimens we found that this species is conspecific with *S.erecta*. This is corresponded with the report of Son et al. (2018).

Son et al. (2018) proposed the duplicate of *Put 3302* from K (without barcode) as the lectotype of *S.suborbicularis*. Unfortunately, we have not seen this duplicate. Moreover, they also provided a picture of the lectotype, but we found that this picture is the sheet BM000906322 at BM, not K: the citation of K may therefore be in error. We were able to locate five duplicates of *Put 3302* one deposited at each of ABD [ABDUH:2/914], BK [257647], BM [BM000906322], C [C10005219] and KYO.

#### ﻿11. Strobilanthes esquirolii

Taxon classificationPlantaeLamialesAcanthaceae

H.Lév., Repert. Spec. Nov. Regni Veg. 12: 18. 1913.

FAAAA981-9F0A-5436-BC8A-BD8C4EC78D44

Figs 4 , 10D

Tetragoga esquirolii (H.Lév.) E.Hossain in Notes Roy. Bot. Gard. Edinburgh 32: 410. 1973.
Strobilanthes bombycina J.B.Imlay, Bull. Misc. Inform. Kew 1939(3): 124. 1939. Type: Thailand, Kanchanaburi; Si Sawat [Si Sawat], 14 Jan 1926, *Kerr 10211* (lectotype BM [BM000906285!] designated here; isolectotypes ABD [ABDUH:2/888 image!], BK [257639!], K [K001514899!]), syn. nov.
Strobilanthes leucocephala Craib, Bull. Misc. Inform. Kew 1914(3): 130. 1914. Type: Thailand, Lamphun, Mae Tha [Me Ta], Doi Din Deng, 3 Feb 1912, *Kerr 2317* (lectotype K [K001514901!] designated here; isolectotypes BM [BM000906289!], E [E00133531!], K [K001514900!, K001514902!, K001514903!], TCD!).
Goldfussia leucocephala (Craib) C.Y.Wu ex H.P. Tsui & C.C.Hu in Fl. Reipubl. Popularis Sin. 70: 165. 2002. Type: Based on Strobilanthesleucocephala Craib.

##### Type.

China, Kweichow, de Pa-Bonn a Ting-Chan, 16 Dec1904, *Esquirol 322* (holotype E [E00133561!]).

**Figure 4.**
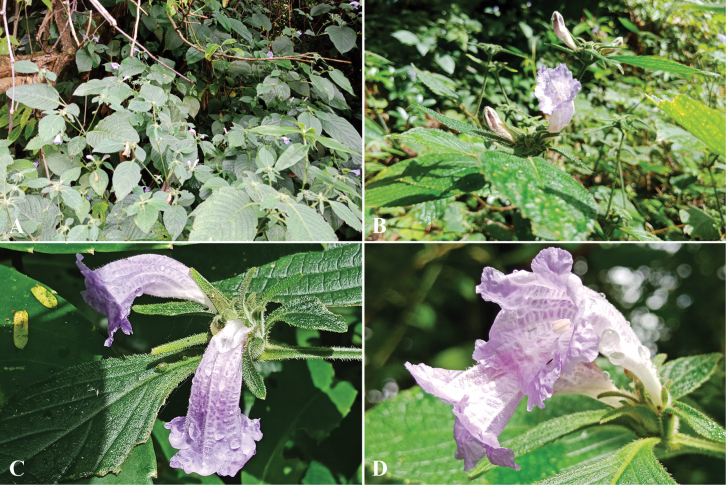
*Strobilanthesesquirolii* H.Lév **A** stem and leaves **B, C** inflorescences **D** corolla, anthers and stigma.

##### Distribution.

Myanmar, China, Thailand, Laos, Vietnam.

##### Ecology.

In hill evergreen forest or mixed deciduous forest with bamboo, limestone granite or sandstone bedrock; 107–2,190 m alt., flowering and fruiting from October to May.

##### Selected specimens examined.

Thailand, Northern: **Mae Hong Son**, Mueang, Tham Pla-Namtok Pha Suea NP, Doi Pha Daeng, 680 m alt., 26 Dec 2012, *Norsaengsri 10016* (BKF, QBG); ibid., Pai, Mae Yen Waterfall, 570 m alt., 15 Jan 1983, *Koyama 32503* (BKF, KYO, L); **Chiang Mai**, Doi Chiang Dao WS, 600–800 m alt., 4 Jan 1954, *Garrett 1427* (K, L2 sheets, P); ibid., 550 m alt., 8 Mar 1965, *Chermsirivathana 298* (BK, BKF); ibid., 1,250–1,425 m alt., 4 Dec 1965, *Hennipman 3216* (BKF, K, KYO, L, P); ibid., 6 Jan 1975, *Geesink 8111* (BKF, K, L); ibid., 1,625 m alt., 5 Nov 1995, *Maxwell 95-1077* (BKF, CMUB, L2 sheets); ibid., 14 Oct 2019, *Kladwong 482* (KKU) & *483* (KKU); ibid., Fang, 750 m alt., 27 Feb 1958, *Sørensen* et al. *1726* (E); ibid., 700–800 m alt., 11 Jan 1975, *Geesink 8217* (AAU, BKF, K, KYO, L, P) & *8220* (BKF, K, L); ibid., Mae Taeng, 1,300 m alt., 23 Nov 2001, *Maxwell 01-626* (BKF, CMUB, L); **Lamphun**, Mae Tha, Doi Din Deng, 3 Feb 1912, *Kerr 2317* (BM, E, K, TCD); **Lampang**, Doi Khun Than NP, 16 Dec 2019, *Balslev* et al. *9910* (AAU); ibid., 25 Oct 2019, *Kladwong 509* (KKU); **Tak**, Umphang, Ban Mae Lamung, 24 Dec 2010, *Suksathan* et al. *5429* (L); **Phitsanulok**, Chat Trakan, Phu Miang, 2 Oct 1968, *Phusomsaeng* et al. *11* (BKF, K, L); ibid., Phu Hin Rong Kla NP, 1,400–1,600 m alt., 10 Dec 1990, *Larsen* et al. *41828* (AAU); North-eastern: **Loei**, Na Haew, Phu Suan Sai NP, 15 May 2008, *Maknoi* & *Srisanga 2293* (QBG); ibid., Phu Kradueng NP, 900–1,300 m alt., 17 Dec 1982, *Koyama* et al. *31205* (BKF, KYO, L); ibid., Phu Luang WS, 1,300 m alt., 14 Mar 1980, *Smitinand s.n.* (BKF); **Bueng Kan**, Phu Wau WS, 197 m alt., 28 Dec 2011, *Norsaengsri* & *Tathana 8698* (BKF, QBG); South-western: **Uthaitani**, Ban Rai, Ban Poo Bon, 300 m alt., 2 Feb 1976, *Maxwell 76-56* (BK, L 2 sheets); **Kanchanaburi**, Si Sawat, Erawan NP, Huai Lam Tam Ton, 580–600 m alt., 26 Nov 1982, *Koyama* et al. *30306* (BKF, KYO, L); without locality, 14 Jan 1926, *Kerr 10211* (ABD, BK, BM, K).

##### Preliminary conservation status assessment.

This species has an Extent of Occurrence (EOO) of 195,058.419 km^2^ and an Area of Occupancy (AOO) of 68.000 km^2^ and is assessed as Least Concern (LC) following IUCN (2022).

##### Notes.

*Strobilanthesesquirolii* resembles *S.brandisii* and *S.consors* in having a densely white tomentose indumentum on bracts, bracteoles and calyx, but it can be distinguished from both in having sulcate stems, oblong-lanceolate bracts. In addition, *S.esquirolii* is also different by dark green stems and bracts.

Craib (1914) described *S.leucocephala* based on *Kerr 2317* which has seven duplicates, four in K [K001514900, K001514901, K001514902, K001514903] and one in each of BM [BM000906289], E [E00133531] and TCD. K001514901 has more inflorescences and flowers than the others; therefore, we select this specimen as the lectotype.

We have examined the types of *S.bombycina* and *S.esquirolii* and found that they are conspecific because they are densely white tomentose on bracts, bracteoles and calyx and the bracts are oblong-lanceolate. The original protologue of *S.bombycina* was based on *Kerr 10211* (Imlay 1939), this number has four duplicates and one at each of ABD [ABDUH:2/888], BK [257639], BM [BM000906285] and K [K001514899]. The morphological characters of the specimen at BM correspond with the protologue, especially in leaf and fruit size and the sheet also has *Imlay*’s handwriting as follows: “*Strobilanthesbombycina* Imlay Type no.”. Therefore, we select the sheet BM000906285 as the lectotype.

#### ﻿12. Strobilanthes falconeri

Taxon classificationPlantaeLamialesAcanthaceae

T.Anderson, J. Linn. Soc., Bot. 9: 484. 1867.

975AE120-6D42-580E-BD38-0154B9C19C5B

Fig. 10D

##### Type.

Myanmar, Moulmain, 27 Feb 1849, *Falconer 423* (lectotype CAL [CAL0000019638 image!] designated here; isolectotypes CAL [CAL0000019639 image!], K [K000882995!]).

##### Distribution.

Myanmar, Thailand.

##### Ecology.

In evergreen forest; 250–1,300 m alt., flowering and fruiting from October to May.

##### Selected specimens examined.

Thailand, Northern: **Mae Hong Son**, Khun Yuam, Mae Yuam Noi, 800 m alt., 24 Mar 2009, *Pongamornkul 2579* (QBG); ibid., Mae Sariang, Mae Bow, 1,125 m alt., 2 Mar 1991, *Maxwell 91-212* (AAU-2 sheets, E, L); ibid., Sob Moei, 900 m alt., 29 Apr 2014, *Pongamornkul 4165* (QBG); **Chiang Mai**, Doi Inthanon NP, Doi Pha Tang, 1,300 m alt., 18 Jan 2009, *Niyomdham & Puudjaa 8356* (BKF); Tak, Umphang, Thung Yai Naresuan East WS, 22 Dec 2011, *Watthana & La-ongsri 4100* (QBG); South-western: **Kanchanaburi**, Sangklaburi, Khao Leam NP, 250 m alt., 16 Dec 2005, *Poopath 421* (BKF-2 sheets); ibid., Khao Yai, 800–900 m alt., 2 Apr 1968, *van Beusekom & Phengklai 302* (AAU, BKF, E, K, L); Central: **Nakhon Nayok**, Khao Yai NP, 1,170 m alt., 9 Oct 1979, *Shimizu* et al. *18097* (KYO-2 sheets); ibid., 800 m alt., 29 Jan 2008, *Maxwell 08-17* (QBG, CMUB, L).

##### Preliminary conservation status assessment.

This species has an Extent of Occurrence (EOO) of 69,009.843 km^2^ and an Area of Occupancy (AOO) of 36.000 km^2^ and is assessed as Least Concern (LC) following IUCN (2022).

##### Notes.

*Falconer 423* was mentioned in the original protologue of *S.falconeri* (Anderson 1867). There are three duplicates two at CAL [CAL0000019638, CAL0000019639] and one at K [K000882995]. All the specimens were labelled as “*Strobilanthesfalconeri* T.Anderson” in *Anderson*’s handwriting. The sheet CAL0000019638 has more leaves, inflorescences and fruits. Therefore, we select this specimen as the lectotype.

This species was formerly known only from Myanmar, but is now known from the Northern, South-Western and Central floristic regions of Thailand.

#### ﻿13. Strobilanthes graminea

Taxon classificationPlantaeLamialesAcanthaceae

J.B.Imlay, Bull. Misc. Inform. Kew 1939(3): 116. 1939.

E5233546-27DD-5A11-AE41-F3477D5BC415

Fig. 11A

Gutzlaffia graminea (J.B.Imlay) Bremek., Verh. Kon. Ned. Akad. Wetensch., Afd. Natuurk., Sect. 2. 41(1): 155. 1944.

##### Type.

Thailand, Tak, Khao [Kao] Hua Mod, 12 June 1922, *Kerr 6118* (holotype BM [BM001046226!]; isotype BK [257641!]).

##### Distribution.

Endemic to Thailand.

##### Ecology.

In open limestone hill; 300–933 m alt., flowering and fruiting from May to August.

##### Selected specimens examined.

Thailand, Northern: **Tak**, Umphang, Doi Hua Mod [Kao Hua Mod], 12 June 1922, *Kerr 6118* (BK, BM); ibid., 800 m alt., 1 May 2006, *Watthana 1970* (CMUB); ibid., 900 m alt., 27 May 2008, *Pooma* et al. *6995* (BKF); ibid., 800 m alt., 2 May 20011, *Watthana 3805* (QBG); ibid., 933 m alt., 18 July 2015, *Phaosrichai 205* (QBG); **Kamphaeng Phet**, Mae Wong NP, 11 July 1999, *Chayamarit* et al. *1795* (BKF); South-western: **Kanchanaburi**, Sangkhla Buri, Nong Lu, Ban Dan Chedi, Khao Condo, 358 m alt., 25 Aug 2010, *Chamchumroon* et al. *4812* (BKF); ibid., Thong Pha Phum, along route 323, 4 km NW from Thong Pha Phum, 240 m alt., 29 Nov 1982, *Koyama* et al. *30473* (BKF, KYO); ibid., 25 Jan 1983, *Koyama* et al. *32887* (BKF, KYO-2 sheets).

##### Preliminary conservation status assessment.

This species has an Extent of Occurrence (EOO) of 3,865.766 km^2^ and an Area of Occupancy (AOO) of 16.000 km^2^ and is assessed as Endangered (EN), B1 a, b (i, ii, iii) following IUCN (2022). This species grows on open limestone hills and is only recorded from a few records. The changes of the habitat through increasing droughts and fires are likely to lead to decline of *S.graminea*.

##### Notes.

*Strobilanthesgraminea* resembles *S.aprica* (Hance) T.Anderson and *S.hypomalla* Benoist in having two exserted stamens, but the fruit of *S.graminea* has 8 seeds whereas there are 4 seeds in *S.aprica* and *S.hypomalla*.

#### ﻿14. Strobilanthes hypomalla

Taxon classificationPlantaeLamialesAcanthaceae

Benoist, Bull. Mus. Natl. Hist. Nat. 27: 543. 1921.

552241FD-8351-5A34-A008-9EB34894FADB

Fig. 11A

##### Type.

Vietnam, Dalat, 27 Nov 1911, *Lecomte & Finet 1524* (lectotype P [P00218435 image!] designated by Kladwong and Chantaranothai 2022, pg. 182).

##### Distribution.

Thailand, Laos, Vietnam.

##### Ecology.

In dipterocarp forest, sandstone bedrock; 340–492 m alt., flowering and fruiting December.

##### Specimens examined.

Thailand, North-eastern: **Bueng Kan**, Phu Lang Ka NP, 492 m alt., 26 Nov 2017, *Suddee* & *Puudjaa 5333* (BKF); ibid., Phu Wua WS, 340 m alt., 15 Oct 2016, *Suddee et al. 5561* (BKF); ibid., trails to Tham Noi Waterfall, 1 Dec 2020, *Kladwong* et al. *539* (KKU).

##### Preliminary conservation status assessment.

This species has an Extent of Occurrence (EOO) of 10,177.798 km^2^ and an Area of Occupancy (AOO) of 16.000 km^2^ and is assessed as the Endangered (EN), B1 a, b (i, ii, iii) following IUCN (2022). This species grows on sandy soil in dipterocarp forest and is only recorded from a few records. The changes of the habitat through increasing droughts and fire are likely to lead to the decline of *S.hypomalla*.

##### Notes.

*Strobilantheshypomalla* resembles *S.aprica*, but differs in having greenish or yellowish green stems, linear-lanceolate leaf shape, and the outside of the corolla is pubescent. Moreover, the pollen of *S.hypomalla* is prolate or subprolate with a 3-colporate aperture and longitudinal spinose ribs on the exine sculpturing as opposed to 3-cryptoaperturate and with short conical spines over the exine in *S.aprica* (Kladwong and Chantaranothai 2022). According to the protologue of *S.hypomalla*, the corolla was described as glabrous outside but the specimens from Thailand show that it is pubescent. Further research based on more specimens is needed to comprehend this variation.

#### ﻿15. Strobilanthes kerrii

Taxon classificationPlantaeLamialesAcanthaceae

Craib, Bull. Misc. Inform. Kew 1912(6): 267. 1912.

38136B72-8EF6-55CC-A995-80A4576A0684

Figs 5 , 11A

Goldfussia kerrii (Craib) Bremek., Verh. Kon. Ned. Akad. Wetensch., Afd. Natuurk., Sect. 2. 41: 231. 1944.
Goldfussia lanuginosa Bremek., Dansk Bot. Ark. 23: 276. 1966. Type: Thailand, Kawng San, 22 Jan 1964, *Hansen 10872* (holotype C n.v.; isotypes L [L0002856!], U [U0000027!]).

##### Type.

Thailand, Phrae [Phrea], Huai [Hue] Kamin, 18 Feb 1910, *Kerr 988* (lectotype K [K001514920!] designated here; isolectotypes BM [BM000793163!], E [E00136697!], K [K001514921!, K001514922!], TCD!).

**Figure 5.**
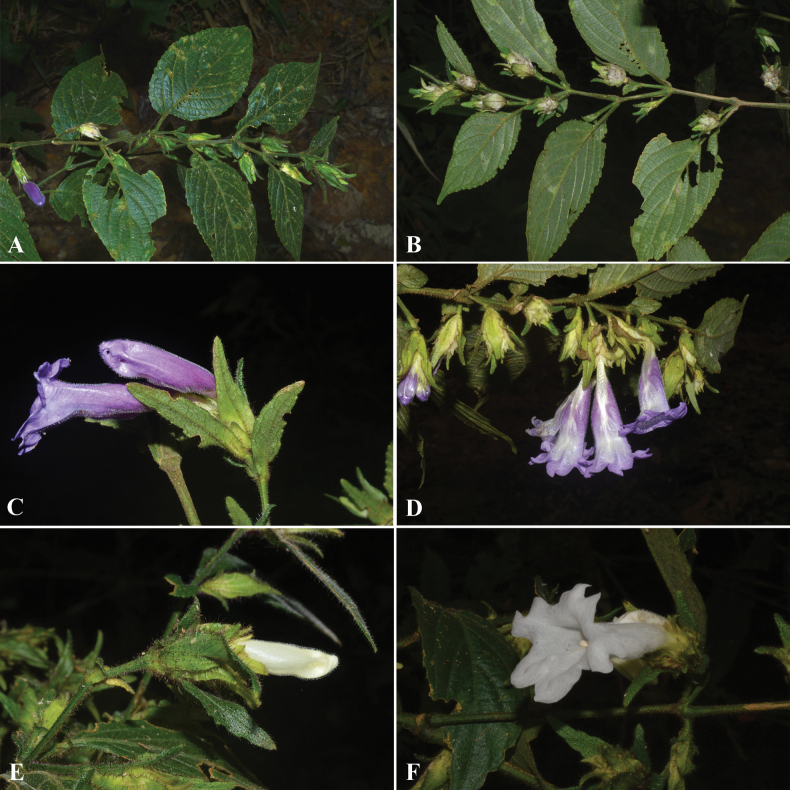
*Strobilantheskerrii* Craib **A** adaxial surface of leaves **B** abaxial surface of leaves **C–F** inflorescences and corolla.

##### Distribution.

Endemic to Thailand.

##### Ecology.

By stream bank in dry evergreen forest and hill evergreen forest; 160–1,800 m alt., flowering and fruiting from September to March.

##### Selected specimens examined.

Thailand, Northern: **Mae Hong Son**, Pai, Mueang Sroi Waterfall, 800 m alt., 17 Jan 1983, *Koyama* et al. *32647* (K, KYO); **Chiang Mai**, Doi Chiang Dao WS, 1,500–1,800 m alt., 27 Oct 1979, *Shimizu* et al. *20918* (BKF, L) *& 20938* (BKF, KYO, L); ibid., Kawng San, 1,150 m alt., 22 Jan 1964, *Hansen 10872* (L, U); **Nan**, Doi Phu Kha NP, 1,510 m alt., 4 Dec 1999, *Srisanga 1233* (BKF, QBG); **Lamphun**, Li, Mae Ping NP, Ko Luang Waterfall, 500 m alt., 23 Jan 2017, *Pooma & Pattharahirantricin 7966* (BKF); **Phrae**, Huai Kamin, ca. 300 m alt., 18 Feb 1910, *Kerr 988* (BM, E, K-3 sheets, TCD); **Tak**, Mae Sot, Inthanin Cave Temple, 26 Dec 2010, *Suksathan* et al. *5447* (L); **Sukhothai**, Kirimat, Ramkhamhaeng NP, 275 m alt., 27 Jan 1995, *Maxwell 95-26* (BKF, CMUB, L); ibid., Srichatchanalai NP, Tham Thara Wasan, 160 m alt., 17 Nov 2014, *Norsaengsri 11509* (QBG). North-eastern: **Loei**, Na Haew, Phu Suan Sai NP, 3 Sept 2008, *Maknoi 2792* (BKF, QBG); ibid., Phu Luang WS, 19 Nov 2019, *Kladwong* et al. *505 & 506* (KKU).

##### Preliminary conservation status assessment.

This species has an Extent of Occurrence (EOO) of 80,801.690 km^2^ and an Area of Occupancy (AOO) of 52.000 km^2^ and is assessed as Least Concern (LC) following IUCN (2022).

##### Notes.

*Strobilantheskerrii* has many characteristics in common with *S.capitata* and *S.speciosa*, especially the leaf and bract shapes and inflorescence type. However, it can be distinguished due to the presence of rigid dark red trichomes on stems, petiole and peduncle that are absent in *S.capitata* and *S.speciosa*. *Strobilantheskerrii* was treated as a synonym of *S.speciosa* (Bennett and Scotland 2003), but now we conclude that it should be regarded as species in its own right.

This species was described by Craib (1912) based on *Kerr 988* which has five duplicates, three of them were deposited at K [K001514920, K001514921, K001514922] and each one kept at BM [BM000793163], E [E00136697] and TCD. All are in good shape, but the sheet K001514920 has more mature leaves and flowers. Therefore, we select this specimen as the lectotype.

#### ﻿16. Strobilanthes paniculata

Taxon classificationPlantaeLamialesAcanthaceae

(Nees) Miq., Fl. Ned. Ind. 2: 802. 1858.

88C74896-A450-54A7-B77B-5DF5599ED733

Fig. 11B

Goldfussia paniculata Nees in Compan. Bot. Mag. 2: 313. 1837. Type: Indonesia, Java, *Hooker s.n.* (lectotype E-GL [E00749036!] designated by Wood 1994a, pg. 112).
Microstrobilus paniculatus (Nees) Bremek., Verh. Kon. Ned. Akad. Wetensch., Afd. Natuurk., Sect. 2. 41(1): 250. 1944. Type: Based on Goldfussiapaniculata Nees.
Strobilanthes deminuta S.Moore in J. Bot. 63: 78. 1925. Type: Indonesia, South Sumatra, Krui, 1880, *Forbes 1929* (lectotype BM [BM0007933206!] designated by Bennett and Scotland 2003, pg. 46; isolectotype L [L0614259!]).
Strobilanthes subcapitata C.B.Clarke in J.D.Hooker, Fl. Brit. India 4: 455. 1884. Type: Myanmar, Tenasserim, *Helfer 6111* (lectotype K [K000883112!] designated here; isolectotypes K [K000883113!], P [P00719448 image!]).
Strobilanthes microcephala Benoist, Bull. Soc. Bot. France 80: 730. 1934. Type: Laos, Bolaven [Boloven], *Poilane 15853* (lectotype P [P00719373 image!] designated here; isolectotypes A [A00286779 image], K [K001514870!, K001514871!], P [P00719376 image!]).

##### Type.

Based on *Goldfussiapaniculata* Nees

##### Distribution.

Myanmar, Thailand, Laos, Indonesia.

##### Ecology.

Near stream in evergreen forest or bamboo forest; 10–1,000 m alt., flowering and fruiting from November to March.

##### Selected specimens examined.

Thailand, Peninsular: **Chumphon**, Lang Suan, 14 Feb 1927, *Kerr 11939* (BK, BM, K, KYO); ibid., 100 m alt., 1 Mar 1927, *Kerr 12172* (BK, BM, K); ibid., Siep Yuan, 10 m alt., 20 Dec 1928, *Kerr 16236* (BK, BM, K); **Ranong**, Kaper, 10 m alt., 17 Feb 1929, *Kerr 16707* (BK, BM, K); ibid., Khao Pawta Luang Kaeo, 400–1,000 m alt., 27 Feb 1983, *Koyama* et al. *33815* (BKF, K, KYO-3 sheets, L); ibid., Klong Naka WS, 80 m alt., 17 Nov 1973, *Santisuk 593* (BKF-3 sheets); ibid., 23 Nov 1974, *Indrapong 39* (BKF); ibid., 30–50 m alt., 8 Dec 1979, *Shimizu* et al. *26398* (BKF, KYO-2); ibid., 30–230 m alt., 6–7 Jan 1990, *Hoover 5072* (E) *& 5429* (E); ibid., Kra Buri, 29 Feb 1968, *Vacharapong 186* (BK) *& 190* (BK); **Surat Thani**, Klong Sok, 14 Feb 1975, *Damrongsak 159* (BKF).

##### Preliminary conservation status assessment.

This species has an Extent of Occurrence (EOO) of 1,739,203.963 km^2^ and an Area of Occupancy (AOO) of 28.000 km^2^ and is assessed as Least Concern (LC) following IUCN (2022).

##### Notes.

*Strobilanthespaniculata* resembles *S.paniculiformis* J.R.I.Wood in having a panicle of capitate inflorescences and glabrous bracts. It differs in having the obovate bracts and linear-obovate bracteoles vs. the ovate-orbicular bracts and obovate to narrowly obovate bracteoles in *S.paniculiformis*.

*Helfer*’s collection was cited in the protologue of *S.subcapitata* (Clarke 1884). This collection has three duplicates. Two of which were deposited at K [K000883112, K000883113] and one housed at P [P00719448]. Both the duplicates at K have *Clarke*’s handwriting as follows: “*Strobilanthessubcapitata* Clarke”: but K000883112 has more mature leaves. Therefore, we select this specimen as the lectotype.

The original protologue of *S.microcephala* was based on *Poilane 15853* (Benoist 1934). This collection has five duplicates one at A [A00286779], two at K [K001514870, K001514871] and two at P [P00719373, P00719376]. P00719373 has more mature leaves and inflorescences; therefore, it is selected as the lectotype. The others are isolectotypes.

#### ﻿17. Strobilanthes paniculiformis

Taxon classificationPlantaeLamialesAcanthaceae

J.R.I.Wood, Kew Bull. 61: 10. 2006.

4CAB1271-463E-5D56-90BE-3465FBBE9D47

Figs 9A , 11C

##### Type.

India, Naga Hills, 1887, *Clarke 40945* (holotype K [K000545689!]; isotype K [K000545690!]).

##### Distribution.

India, Myanmar, Thailand.

##### Ecology.

In hill evergreen forest or open plateau; 1,200–2,285 m alt., flowering and fruiting from October to December.

##### Selected specimens examined.

Thailand, Northern: **Chiang Mai**, Fang, Doi Pha Hom Pok NP, 2,285 m alt., 10 Nov 2012, *Chamchumroon 5541* (BKF); **Phitsanulok**, Phu Hin Rong Kla NP, 1,400–1,600 m alt., 10 Dec 1990, *Larsen* et al. *41827* (AAU, P); ibid., 1,200 m alt., 11 Dec 1990, *Larsen* et al. *41870* (AAU); ibid., 1,300 m alt., 14 Oct 1998, *Suksathan 1302* (QBG-2 sheets); North-eastern: **Loei**, Phu Kradueng NP, 1,150–1,250 m alt., 1 Nov 1984, *Murata* et al. *42539* (BKF, L).

##### Preliminary conservation status assessment.

This species has an Extent of Occurrence (EOO) of 12,195.648 km^2^ and an Area of Occupancy (AOO) of 12.000 km^2^ and is assessed as Vulnerable (VU), B1 a, b (i, ii, iii) following IUCN (2022). This species grows on the open plateau of evergreen mountains and is recorded from only a few collections. The changes of the habitat through increasing droughts and fire might lead to causing decline of *S.paniculiformis*.

##### Notes.

*Strobilanthespaniculiformis* was formerly known from the Naga Hills of Eastern India was also recently recorded in the Kachin State and Sagaing Region of Myanmar (Wood et al. 2022) but is now seen to have a wider distribution stretching into the Northern and North-Eastern floristic regions of Thailand.

#### ﻿18. Strobilanthes phengklaii

Taxon classificationPlantaeLamialesAcanthaceae

Kladwong & Chantar. sp. nov.

C221826B-120C-56BF-BD43-A4F583BC1496

urn:lsid:ipni.org:names:77344994-1

Figs 6 , 7 , 11B

##### Type.

Thailand, Chaiyaphum, Phu Khieo WS, Oct 1999, *Phengklai et al. 12261* (holotype BKF [SN127785!]; isotypes BKF [SN143321!, SN127784!]).

**Figure 6.**
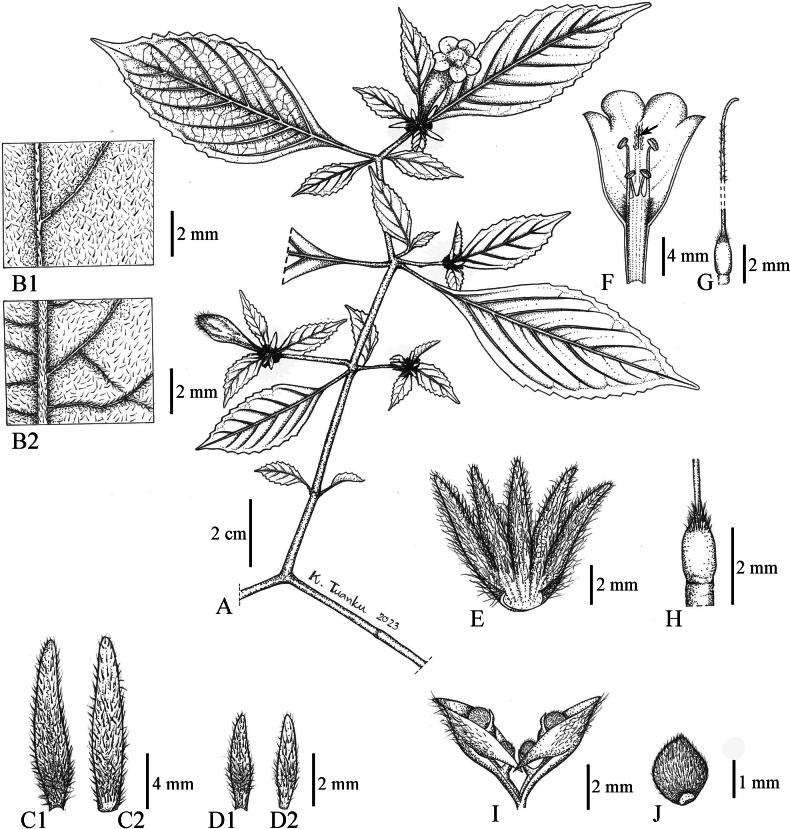
*Strobilanthesphengklaii* Kladwong & Chantar. **A** stem, leaves and inflorescences **B** adaxial surface of leaf (**B1**) and abaxial surface of leaf (**B2**) **C** outer surface of bract (**C1**) and inner surface of bract (**C2**) **D** outer surface of bracteole (**D1**) and inner surface of bracteole (**D2**) **E** calyx **F** corolla, stamens and rugula and trichomes retaining the style (arrow) **G** pistil, style and stigma **H** ovary **I** fruit **J** seed. **A**–**H** drawn from *Phengklai* et al. *12261* (BKF: holotype), **I**–**J** drawn from *Tagawa* et al. *1076* (BKF). Drawn by K. Tuanku.

**Figure 7.**
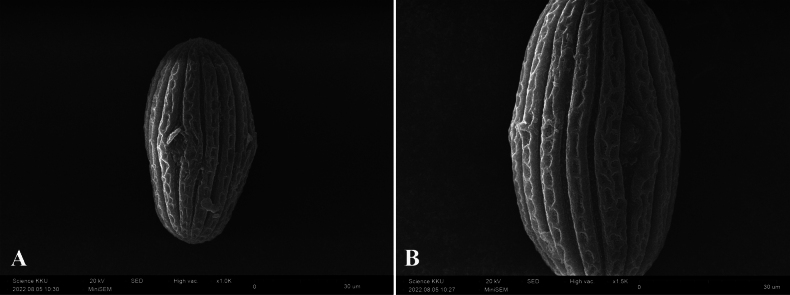
Scanning electron microscope micrographs of pollen of *S.phengklaii* Kladwong & Chantar., equatorial view **A** shape **B** exine sculpturing, from *Phengklai* et al. *12261* (BKF).

##### Diagnosis.

Similar to *S.brandisii* T.Anderson and *S.esquirolii* H.Lév. in having single capitate inflorescences, sessile or subsessile and densely white sericeous bracts but differs in having lanceolate or linear-lanceolate bracts and yellow corolla vs. spathulate bracts and purple corolla in *S.brandisii* and oblong-lanceolate bracts and violet corolla in *S.esquirolii*. The morphological difference among *S.brandisii*, *S.esquirolii* and *S.phengklaii* are presented in Table 2.

**Table 2.** Morphological differences between *Strobilanthesphengklaii* and similar species.

Characters	*S.brandisii*	*S.esquirolii*	*S.phengklaii*
Leaf blade	lanceolate or ovate-lanceolate	ovate-elliptic to ovate-elliptic	lanceolate to oblong-lanceolate
Bract shape	spathulate	oblong-lanceolate	lanceolate or linear-lanceolate
Bracteoles	linear	oblanceolate	linear
Corolla	purple	violet	yellow
Long filaments	hairy	hairy	glabrous
Capsule	ovoid	oblongoid	fusiform

##### Description.

***Herbs*** up to 40 cm tall, perennial, erect or decumbent, anisophyllous. ***Stems*** 4-angled, sulcate or subterete when mature, pubescent or glabrescent. ***Leaves*** petiolate; blades lanceolate to oblong-lanceolate, 1.8–10 × 0.6–3.5 cm, hairy on adaxial surface, pubescent on abaxial surface, lateral veins 3–7 pairs, prominent on both surfaces, apex attenuate to caudate, base attenuate, and decurrent onto petiole, margin serrate or crenate, ciliate; petiole 0.3–2.5 cm long, pubescent. ***Inflorescences*** terminal capitate, 3–5-flowered; peduncle absent; bracts lanceolate or linear-lanceolate, 5–13 × 2–4 mm, persistent, the outer one longer than the inner, white sericeous on both surfaces, the upper part sparsely hispid, apex obtuse, margin entire or obscurely serrate, base sessile, ciliate; bracteoles linear, 3–5 × ca. 0.8 mm, white sericeous on both surfaces. ***Calyx*** 5-lobed; lobes linear, 4–5 × 0.5 mm, subequal, apex acute, white sericeous on both surfaces. ***Corolla*** yellow, funnel shaped, 2–4 cm long, densely white sericeous on top at bud, sparsely pubescent at anthesis, glabrous inside except hairs retaining style; tube yellow, cylindric for 4–6 mm long; mouth 1–1.5 cm wide; lobes 5, ovate, 3–4.5 × 3.5–5 mm, apex obtuse. ***Stamens*** 4, included, didynamous; short filaments 2 mm long, long filament 3–5 mm long, all filament straight and glabrous; anther thecae ca. 1.5 mm long, white, without spur; pollen 3-colporate, prolate or subprolate in equatorial view, circular in polar view, polar range 51–67 μm, equatorial range 31–41 μm; ectoapertures fusiform; exine divided into longitudinal ribs, each rib with a coarse ladder-like reticulum. ***Ovary*** ellipsoid, 2 mm long, densely white sericeous at apex; style 2.5–3 cm long, puberulous. ***Capsule*** fusiform, 5–7 × 3–4 mm, white sericeous, 4-seeded. ***Seeds*** ovate in outline, ca. 1.8× ca. 1.5 mm, hairy.

##### Distribution.

Endemic to Thailand.

##### Ecology.

Common in shaded areas in dry evergreen forest; ca. 600 m alt., flowering and fruiting from October to December.

##### Etymology.

Named in honour of Dr Chamlong Phengklai, a senior botanist at the Forest Herbarium (BKF) who collected the type specimens of *S.phengklaii*.

##### Additional specimens examined

**(*paratypes*)**: Thailand, North-eastern: **Loei**, Phu Luang WS, from Ban Na Luang to north ride ca. 600 m alt., 3 Dec 1965, *Tagawa* et al. *1076* (BKF, KYO, L [L2842098]).

##### Preliminary conservation status assessment.

This species is only known from two populations suggesting that this species is endemic to the north-eastern floristic region of Thailand. It is assessed as Data Deficient (DD) following IUCN (2022). However, *S.phengklaii* was legally collected from a protected area and it is recorded as common in the locality. *Strobilanthesphengklaii* has a few records. The changes of the habitat through increasing droughts and fire is likely to lead to the decline of this species. More field work is needed to assess the conservation status of *S.phengklaii*.

#### ﻿19. Strobilanthes phyllocephala

Taxon classificationPlantaeLamialesAcanthaceae

J.R.I.Wood & Scotland, Nat. Hist. Bull. Siam Soc. 62(1): 31. 2017.

B485CB48-2620-5F59-899C-578EFCBCC015

Fig. 11C

##### Type.

Thailand: Phetchaburi, Kaeng Krachan NP, 6 Aug 1995, *Larsen* et al. *45466* (holotype K [K000224872!]; isotype AAU!).

##### Distribution.

Endemic to Thailand.

##### Ecology.

Near stream in evergreen forest or bamboo forest; 10–1,000 m alt., flowering and fruiting from November to March.

##### Selected specimens examined.

Thailand, South-western: **Phetchaburi**, Kaeng Krachan NP, 400–600 m alt., 6 Aug 1995, *Larsen* et al. *45466* (AAU, K); ibid., 840 m alt., 24 Oct 2013, *Tagane* et al. *2132* (BKF).

##### Preliminary conservation status assessment.

This species is only known from its type locality and is assessed as Data Deficient (DD) following IUCN (2022). More field work needed to assess the conservation status of *S.phyllocephala*.

##### Notes.

*Strobilanthesphyllocephala* resembles *S.falconeri*. It differs in having the ovate or ovate-elliptic leaves vs. elliptic-lanceolate or lanceolate leaves in *S.falconeri*. The apex of bracteoles of *S.phyllocephala* is obtuse vs. acute in *S.falconeri*.

#### ﻿20. Strobilanthes phyllostachya

Taxon classificationPlantaeLamialesAcanthaceae

Kurz, J. Asiat. Soc. Bengal, Pt. 2, Nat. Hist. 40(1): 75. 1871.

8EB431C0-FF78-5315-B7FE-97D37DC6886B

Figs 8 , 11C

Sericocalyx phyllostachyus (Kurz) Bremek., Verh. Kon. Ned. Akad. Wetensch., Afd. Natuurk., Sect. 2. 41(1): 163. 1944.

##### Type.

Myanmar, Bago Region [Beeliz], *Brandis s.n.* (syntype K! [without barcode]).

**Figure 8.**
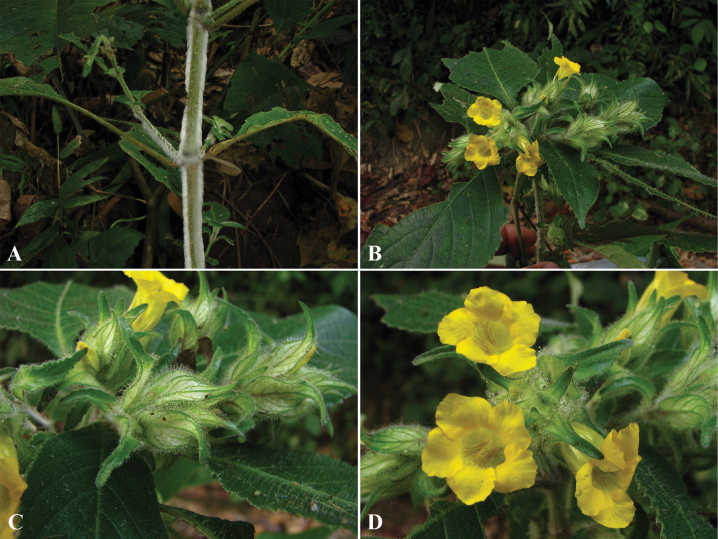
*Strobilanthesphyllostachya* Kurz **A** stem **B** leaves and inflorescences **C** bracts **D** corolla. Photo by P. Suksathan.

##### Distribution.

Myanmar, Thailand.

##### Ecology.

Near stream in evergreen forest or bamboo forest; 10–1,000 m alt., flowering and fruiting from November to March.

##### Selected specimens examined.

Thailand, Northern: **Mae Hong Son**, Mae Sariang, 400 m alt., 21 Feb 1982, *Wongprasert 6* (BKF-2 sheets, K, L, P); ibid., Huai Ngae, 350 m alt., 14 Feb 1971, *Smitinand & Boonkird 11422* (BKF); 450 m alt., 13 Jan 1988, *Santisuk 6668* (BKF-2 sheets); ibid., Salawin WS, Huai Ka Han, 500 m alt., 23 Mar 2006, *Watthana & Wongnak 1860* (QBG); ibid., Sob Moei, 950 m alt., 24 Jan 2015, *Pongamornkul 4782* (QBG); ibid., Mae Ngao NP, 13 Jan 2015, *Tanming 757* (QBG); **Tak**, Mae Ngo NP, 395 m alt., 23 Dec 2010, *Suksathan* et al. *5336* (L); ibid., Tha Song Yang, 22 Mar 2006, *Pooma* et al. *6226* (AAU, BKF-2 sheets, QBG); South-western: **Kanchanaburi**, Thong Pha Phum, 14 Dec 1993, *Parinya* et al. *364* (BK); ibid., Wangka, Kwae Noi River Basin, 150 m alt., 13 May 1949, *Kostermans 412* (K).

##### Preliminary conservation status assessment.

This species has an Extent of Occurrence (EOO) of 10,101.500 km^2^ and an Area of Occupancy (AOO) of 28.000 km^2^ and is assessed as Vulnerable (VU), B1 a, b (i, ii, iii) following IUCN (2022). This species grows near streams in evergreen forest or bamboo forest with a few records. The changes of the habitat through increasing droughts and fire are likely to lead to the decline of *S.phyllostachya*.

##### Notes.

*Strobilanthesphyllostachya* is distinguishable from *S.squalens* S. Moore by its elliptic bract and hairlessness on the outside of the corolla. This species was formerly only known from Myanmar (Kurz 1871; Wood et al. 2022), but is now newly recorded from the Northern and Southwestern floristic regions of Thailand.

#### ﻿21. Strobilanthes serpens

Taxon classificationPlantaeLamialesAcanthaceae

(Nees) J.R.I.Wood & Scotland, Kew Bull. 77: 834. 2021.

752DA055-69CA-57D3-8397-D748B10E86FD

Fig. 11C

Ruellia serpens Nees, Prodr. [A. P. de Candolle] 11: 145. 1847. Type: Indonesia [insular Java], Tjiserae, 1826, *Blume s.n.* (holotype GZU [GZU000250312 image!]; isolectotypes B [B101182406 image!], L [L0065807!]).
Hemigraphis serpens (Nees) Boerl., Handl. Fl. Ned. Ind. 2(2): 658. 1899. Type: Based on Ruelliaserpens Nees.
Hemigraphis hispidula Craib, Bull. Misc. Inform. Kew 1913(6): 203. 1913. Type: Thailand, Nan, 21 Feb 1912, *Kerr 2383a* (lectotype K [K000882585!] designated here; isolectotype E [E00273431!]), syn. nov.
Sericocalyx hispidulus (Craib) Bremek., Verh. Kon. Ned. Akad. Wetensch., Afd. Natuurk., Sect. 2. 41(1): 163. 1944. Type: Based on Hemigraphishispidula Craib.
Hemigraphis ridleyi C.B.Clarke, J. Asiat. Soc. Bengal, Pt. 2, Nat. Hist. 74(3): 652. 1908. Type: Malaysia, Pahang, Jerantut, Kuala Tembeling, Aug 1891, *Ridley 2180* (holotype SING [SING0027181 image!]).

##### Type.

Based on *Ruelliaserpens* Nees

##### Distribution.

Thailand, Malaysia, Singapore, Indonesia.

##### Ecology.

In grass thickets and meadows or by streams under the shade of mixed deciduous and evergreen forests; 10–1,250 m alt., flowering and fruiting from June to May.

##### Selected specimens examined.

Thailand, Northern: **Chiang Rai**, Doi Luang NP, 580 m alt., 3 Nov 2015, *Muangyen 217* (QBG); **Chiang Mai**, Mae Taeng, Pong Dueat, 825 m alt., 19 Nov 1992, *Maxwell 92-742* (CMUB, L); **Nan**, Tha Wang Pha, 500 m alt., 14 Nov 1993, *Larsen* et al. *44347* (AAU); ibid., Huai Mae Sakawn, between Phrae and Nan, ca. 427 m alt, 15 Feb 1912, *Kerr 2383* (E, K); ibid., common in Jungle, 21 Feb 1912, *Kerr 2383a* (E); **Phitsanulok**, Thung Salang Luang NP, 600 m alt., 25 July 1996, *Larsen* et al. *885* (AAU, BKF); Kamphaeng Phet, Klong Klung, 200 m alt., 1 June 1922, *Kerr 6044* (BK, K); North-eastern: **Phetchabun**, Nam Nao NP, 11 June 1964, *Chantanamuck 751* (BK), **Loei**, Na Haew, 1,000 m alt., 26 Apr 1994, *Nanakorn* et al. *3168* (QBG); ibid., Phu Kradueng NP, Phen Phop Mai Waterfall, 1,230 m alt., 4 Sept 1988, *Tsuchiya & Tamura 60534* (BKF); **Khon Kaen**, Chum Phae, Pha Nok Khao, 400 m alt., 26 Nov 1965, *Tagawa 294* (BKF, K, L); ibid., Phu Pha Man NP, Nakarat Cave, 483 m alt., 25 July 2010, *Norsaengsri & Thangson 6950* (QBG); South-western: **Kanchanaburi**, Huai Bankao, 800 m alt., 13 Nov 1971, *van Beusekom* et al. *3758* (BKF, K, L); ibid., Sai Yok, Thung Kang Yang, 5 July 1963, *Larsen 10516* (BKF, L); ibid., Sangkhla Buri, Mueang Cha area, 800 m alt., 8 July 1973, *Maxwell 73-218* (AAU, BK); **Ratchaburi**, Chom Bueng, Ban Baw, 100 m alt., 26 Mar 1975, *Maxwell 75-332* (AAU, BK); ibid., Thung Kang Yang, 350 m alt., July 1963, *Larsen* et al. *10517* (AAU); South-eastern: **Chon Buri**, Si Racha, 15 Nov 1926, *Put 458* (BK, K); **Chanthaburi**, Pong Nam Ron, Khao Soi Dao, 250 m alt., 5 May 1975, *Maxwell 75-485* (AAU); Peninsular: **Chumphon**, Sawi, 9 Sept 1927, *Put 1023* (BK, K); **Ranong**, Kra Buri, Nam Chut, 29 Jan 1927, *Kerr 11704* (BK, K); La-un, 10 m alt., 2 Jan 1929, *Kerr 16493* (BK, K); **Krabi**, Khao Panom Bencha, 24 Oct 1991, *Larsen* et al. *42533* (AAU); **Nakhon Si Thammarat**, Lan Saka, 50 m alt., 25 Apr 1928, *Kerr 15384* (BK); **Trang**, Khao Chong, 200 m alt., 12 Aug 1975, *Maxwell 75-767* (AAU); **Songkhla**, Rattaphum, Boriphat Waterfall, 100–200 m alt., 19 Oct 1991, *Larsen* et al. *42390* (AAU, BKF); ibid., 250 m alt., 16 Aug1984, *Maxwell 84-67* (BKF, L).

##### Preliminary conservation status assessment.

This species has an Extent of Occurrence (EOO) of 399,832.702 km^2^ and an Area of Occupancy (AOO) of 100.000 km^2^ and is assessed as Least Concern (LC) following IUCN (2022).

##### Notes.

*Strobilanthesserpens* differs from *S.hirta* Blume in having petioles which are 2–3.5 cm long, obovate bracts and white anthers. *Strobilantheshirta* has very short or sessile petioles, ovate-elliptic bracts and purplish-red anthers.

*Hemigraphishispidula* was described by Craib (1913). However, after investigation of type and non-type specimens from Thailand, we find that this species has many characteristics in common with *S.serpens*. Therefore, *H.hispidula* is placed as a synonym of *S.serpens*. The original protologue of *H.hispidula* was based on *Kerr 2383* and *Kerr 2383a* (Craib 1913). There are two duplicates of *Kerr 2383* one at E [E00273430] and the other at K [K000882584] and there are two duplicates of *Kerr 2383a* one at E [E00273431] and the other at K [K000882585]. All are in good shape: we designate the sheet K000882585 as the lectotype because it has more mature stems and leaves.

#### ﻿22. Strobilanthes speciosa

Taxon classificationPlantaeLamialesAcanthaceae

Blume, Bijdr. Fl. Ned. Ind. 14: 799. 1826.

920A3F32-52F1-5103-8644-414DA7B7B633

Fig. 11D

Goldfussia speciosa (Blume) Bremek., Verh. Kon. Ned. Akad. Wetensch., Afd. Natuurk., Sect. 2. 41: 227. 1944. Type: Based on Strobilanthesspeciosa Blume.
Strobilanthes apoesensis Hochr., Candollea 5: 228. 1934. Type: Indonesia, Mt. Salak, 7 Mar 1904, *Hochreutiner 101* (syntype G [G00236511 image!]).
Strobilanthes kinabaluensis Stapf, Trans. Linn. Soc. London, Bot. 4: 214. 1894. Type: Malaysia, Sabah, Kadamaian River, *Haviland 1362* (syntype K *fide*Bennett and Scotland 2003, pg. 40).
Goldfussia kinabaluensis (Stapf) Bremek., Verh. Kon. Ned. Akad. Wetensch., Afd. Natuurk., Sect. 2. 41: 229. 1944. Type: Based on Strobilantheskinabaluensis Stapf.
Strobilanthes pierrei Benoist, Bull. Soc. Bot. France 80: 730. 1934. Type: Thailand, Khao [Kow] Luang, Aug 1868, *Pierre s.n.* (lectotype P [P00218442 image!] designated here; isolectotypes A [A00286777 image!], P [P00719405 image!, P00204977 image!], NY [00278319 image!]).

##### Type.

Indonesia, Megamendung, *Blume 1802* (lectotype L [L0537293!] designated by Bennett and Scotland 2003, pg. 39).

##### Distribution.

China, Thailand, Laos, Cambodia, Malaysia, Indonesia.

##### Ecology.

Common in evergreen forest or on rugged limestone area, 100–2,150 m alt. Flowering and fruiting from August to March.

##### Selected specimens examined.

Thailand, Northern: **Mae Hong Son**, Khun Yuam, 1,000 m alt., 20 Nov 2014, *Prommanut & Rattanathip 618* (BK-4 sheets); **Chinag Mai**, Doi Chiang Dao WS, 1,500 m alt., 15 Sept 1967, *Shimizu & Hutoh 10152* (BKF, KYO-2 sheets, L); ibid., Fang, Doi Ang Khang, 1,600 m alt., 17 Nov 1973, *Sadakorn 289* (BK); **Chiang Rai**, Doi Chang, 868 m alt., 28 Nov 2010, *Norsaengsri & Tathana 7365* (QBG); ibid., Mae Sai, 1,350 m alt., 4 Oct 1992, *Banziger 1055* (CMUB, L); **Nan**, Song Khwae, Bo Pra Kang, 657 m alt., 2 Feb 2011, *La-ongsri* et al. *2067* (QBG, PSU); **Lampang**, Wang Nuea, Chae Son NP, 525 m alt., 25 Oct 1995, *Maxwell 95-1006* (BKF, CMUB, L); **Tak**, Doi Muser, 700 m alt., 27 Feb 1987, *Paisooksantivatana 2029-87* (BK); ibid., Phummipol Dam, Dec 1959, *S.N. 675* (BK); **Sukhothai**, Mueang Kao, 4 Nov 1971, *Maxwell 71-677* (AAU, BK); South-western: **Phetchaburi**, Kaeng Krachan NP, 210 m alt., 12 Dec 2002, *Middleton 1588* (BKF, CMUB, E); **Prachuap Kiri Khan**, Kaeng Krachan NP, 260 m alt., 15 Aug 2002, *Middleton 1078* (AAU, BKF, CMUB, E, L); ibid., Huai Yang, 6 Oct 1980, *Put 3229* (BK, K, KYO); Peninsular: **Surat Thani**, Phanom, Chong Lom, Khao Sok NP, 100–150 m alt., 12 Dec 1979, *Shimizu* et al. *27115* (BKF, KYO-2 sheets, L); **Nakhon Si Thammarat**, Kiriwong, 100 m alt., 28 Apr 1928, *Kerr 15420* (BK, BM, K); ibid., 100–700 m alt., 17 Jan 1966, *Tagawa* et al. *4545* (BKF, KYO-2 sheets, L); ibid., Khao [Kow] Luang, Aug 1868, *Pierre s.n.* (A, NY, P-4 sheets).

##### Preliminary conservation status assessment.

This species has an Extent of Occurrence (EOO) of 250,984.816 km^2^ and an Area of Occupancy (AOO) of 64.000 km^2^ and is assessed as Least Concern (LC) following IUCN (2022).

##### Notes.

*Pierre*’s collection was cited in the protologue of *S.pierrei* (Benoist 1934). This collection has five duplicates, there are three at P [P00218442, P00719405, P00204977], one at A [A00286777] and one at NY [00278319]. All specimens bear *Benoist*’s handwriting as follows: “*Strobilanthespierrei* R. Benn.”. However, P00218442 has more leaves and inflorescences; therefore, it is selected as the lectotype. The others are isolectotypes.

#### ﻿23. Strobilanthes squalens

Taxon classificationPlantaeLamialesAcanthaceae

S.Moore, J. Nat. Hist. Soc. Siam 4: 151. 1921.

ABED207B-F59D-5F8A-8047-438F1F6828A0

Figs 9B , 11D

Sericocalyx thailandicus Bremek., Dansk Bot. Ark. Dansk Bot. Ark. 20: 68. 1961. Type: Thailand, Chanthaburi, between Makham and Soi Dao, 100–200 m alt., 14 Jan 1958, *Sørensen* et al. *241* (holotype L [U0000114!]: isotype C [C10005215 image!]), syn. nov.

##### Type.

Vietnam, South Annam, Langbian, Dran, Mar 1918, *Kloss s.n.* (holotype BM [BM000810180!]).

**Figure 9.**
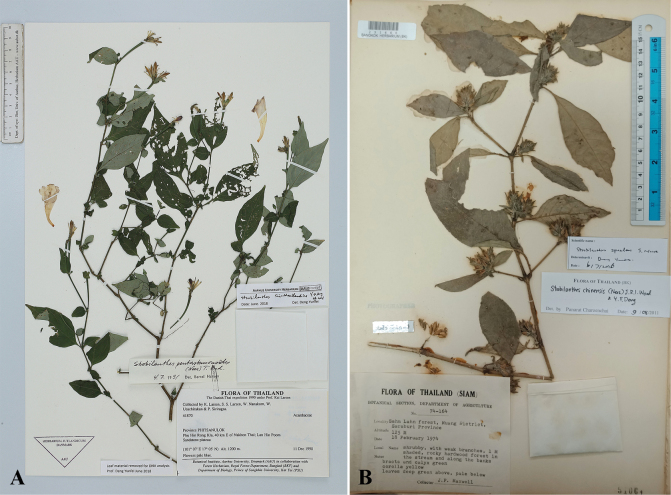
Photographs of dried specimens **A***S.paniculiformis* J.R.I.Wood from *Larsen* et al. *41870* (AAU) **B***S.squalens* S.Moore from *Maxwell 74-164* (BK).

**Figure 10.**
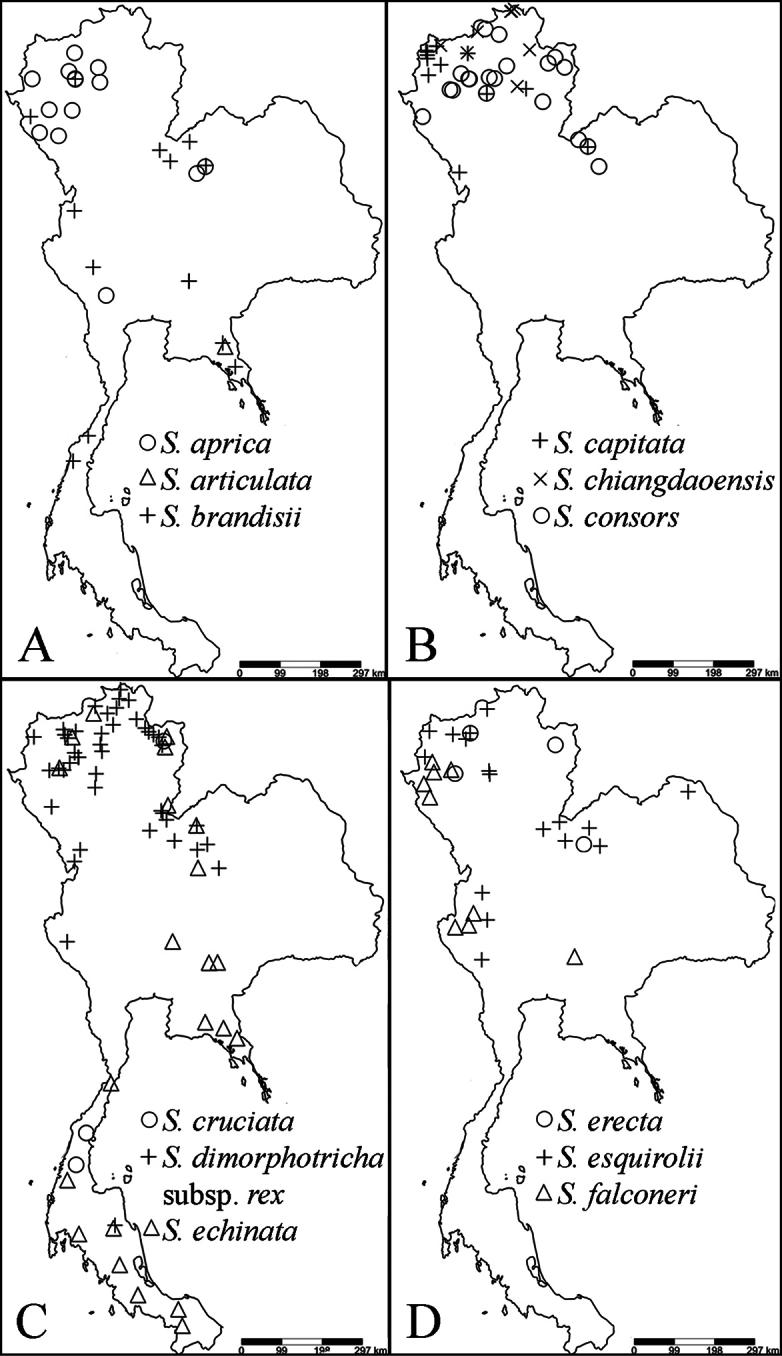
Distribution maps of *Strobilanthes* with capitate inflorescence in Thailand **A***S.aprica* (Hance) T.Anderson, *S.articulata* J.B.Imlay and *S.brandisii* T.Anderson **B***S.capitata* (Nees) T.Anderson, *S.chiangdaoensis* Terao and *S.consors* C.B.Clarke **C***S.cruciata* (Bremek.) Terao, S.dimorphotrichasubsp.rex (C.B.Clarke) J.R.I.Wood and *S.echinata* Nees **D***S.erecta* C.B.Clarke, *S.esquirolii* H.Lév. and *S.falconeri* T.Anderson.

**Figure 11.**
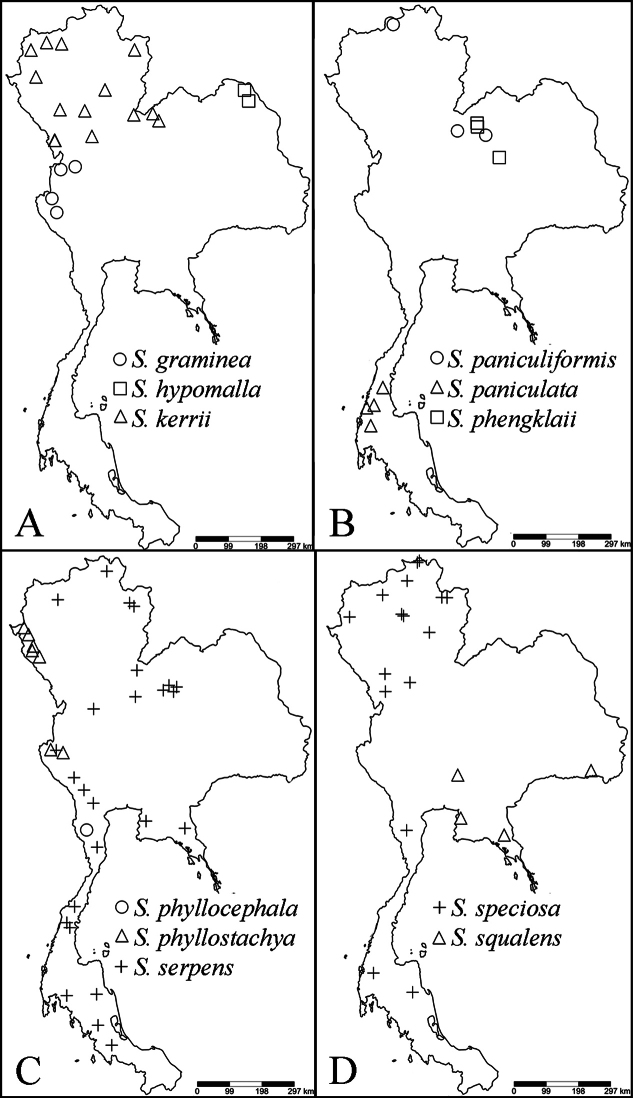
Distribution maps of *Strobilanthes* with capitate inflorescence in Thailand **A***S.graminea* J.B.Imlay, *S.hypomalla* Benoist and *S.kerrii* Craib **B***S.paniculiformis* J.R.I.Wood, *S.paniculata* (Nees) Miq. and *S.phengklaii* Kladwong & Chantar. **C***S.phyllocephala* J.R.I.Wood & Scotland, *S.phyllostachya* Kurz and *S.serpens* (Nees) J.R.I.Wood & Scotland **D***S.speciosa* Blume and *S.squalens* S.Moore.

##### Distribution.

Thailand, Vietnam.

##### Ecology.

Near stream in evergreen forest or bamboo forest; 10–1,000 m alt., flowering and fruiting from November to March.

##### Specimens examined.

Thailand, Eastern: **Si Sa Ket**, Kantharalak, Khao Phra Wihan NP, 400 m alt., 21 Dec 2005, *Pooma* et al. *6036* (BKF); ibid., 200 m alt., 22 Dec 2005, *Pooma* et al. *6091* (BKF, E, L); Central: **Saraburi**, Mueang, Sam Lan Forest, 125 m alt., 18 Feb 1974, *Maxwell 74-164* (AAU, BK); ibid., 100 m alt., 26 Jan 1975, *Geesink & Maxwell 8377* (BKF, L); South-eastern: **Chon Buri**, Sriracha, Nong Kam Kheo, ca. 122 m alt., 1 Dec 1927, *Collins 1832* (BK, BM, K); **Chanthaburi**, between Makham and Soi Dao, 100–200 m alt., 14 Jan 1958, *Sørensen et al. 241* (C, L); ibid., Tap Sai, 200 m alt., 17 Dec 1924, *Kerr 9693* (BK, BM, K); ibid., 200 m alt., 19 Dec 1924, *Kerr 9693A* (BK, BM).

##### Preliminary conservation status assessment.

This species has an Extent of Occurrence (EOO) of 46,083.781 km^2^ and an Area of Occupancy (AOO) of 16.000 km^2^ and is assessed as Least Concern (LC) following IUCN (2022).

##### Notes.

*Strobilanthessqualens* was formerly thought to be an endemic to Southern Vietnam (Baker et al. 1921), but it is now found to occur in the Eastern and Central floristic regions of Thailand.

*Sericocalyxthailandicus* was described by Bremekamp (1961). After investigation of type specimens from Thailand, we find that this species is conspecific with *S.squalens*. Therefore, *S.thailandicus* is placed as a synonym of *S.squalens*.

## Supplementary Material

XML Treatment for Strobilanthes aprica

XML Treatment for Strobilanthes articulata

XML Treatment for Strobilanthes brandisii

XML Treatment for Strobilanthes capitata

XML Treatment for Strobilanthes chiangdaoensis

XML Treatment for Strobilanthes consors

XML Treatment for Strobilanthes cruciata

XML Treatment for Strobilanthes dimorphotricha subsp. rex

XML Treatment for Strobilanthes echinata

XML Treatment for Strobilanthes erecta

XML Treatment for Strobilanthes esquirolii

XML Treatment for Strobilanthes falconeri

XML Treatment for Strobilanthes graminea

XML Treatment for Strobilanthes hypomalla

XML Treatment for Strobilanthes kerrii

XML Treatment for Strobilanthes paniculata

XML Treatment for Strobilanthes paniculiformis

XML Treatment for Strobilanthes phengklaii

XML Treatment for Strobilanthes phyllocephala

XML Treatment for Strobilanthes phyllostachya

XML Treatment for Strobilanthes serpens

XML Treatment for Strobilanthes speciosa

XML Treatment for Strobilanthes squalens

## ﻿Additional information

### Author ORCIDs

Pornchai Kladwong https://orcid.org/0000-0003-3504-7434

Pranom Chantaranothai https://orcid.org/0000-0002-5065-6169

### Data availability

All of the data that support the findings of this study are available in the main text.
